# Research on dust control and ventilation parameter optimization in roadhead mining based on response surface methodology

**DOI:** 10.1371/journal.pone.0338763

**Published:** 2026-09-23

**Authors:** Xiaokun Zhao, Yuting Zhang, Jun Ge, Wencai Wang, Yuhua Bai, Donghui Yang

**Affiliations:** 1 School of Coal Engineering, Shanxi Datong University, Datong, Shanxi, China; 2 The Cultivation Base of Shanxi Key Laboratory of Coal Mine Water Jet Technology and Equipment, Shanxi Datong University, Datong, Shanxi, China; 3 School of Mining and Coal, Inner Mongolia University of Science and Technology, Baotou, Inner Mongolia, China; NED University of Engineering and Technology, PAKISTAN

## Abstract

At fully mechanized heading faces, dust accumulation in roadways poses persistent safety and occupational health risks, particularly under long-forcing, short-exhaust hybrid ventilation conditions, with the return-air-side corners being critical dust accumulation zones. This study aimed to optimize the ventilation system and reduce dust mass concentrations by combining numerical simulations with response surface methodology (RSM). A simplified geometric model was developed in SpaceClaim, and numerical simulations were performed using ANSYS Fluent. Single-factor analysis and a Box--Behnken experimental design were employed to investigate the effects of the exhaust duct inlet angle, exhaust-to-forcing airflow ratio, exhaust duct inlet diameter, and forcing duct outlet diameter. The results showed that the exhaust duct inlet angle and diameter were the dominant factors affecting dust distribution. Through multi-objective optimization, the dust mass concentrations at the lower and upper return-air-side corners were reduced by 58% and 34%, respectively. Validation using a 1:4 scaled-model experimental platform showed that the experimental measurements agreed well with the numerical predictions, with relative errors below 5%. This study developed an integrated research framework combining numerical simulation, response surface optimization, and scaled-model experimental validation to address dust accumulation in the return-air-side corner regions under a long-forcing, short-exhaust hybrid ventilation system. The proposed framework enables the coupled analysis of multiple ventilation parameters and the dual-objective optimization of dust mass concentrations at the upper and lower return-air-side corners, providing a reference for dust control and ventilation parameter optimization under similar operating conditions.

## 1. Introduction

Coal mine dust generation and accumulation pose persistent risks to operational safety and workers’ health. High airborne dust concentrations reduce underground visibility, accelerate equipment wear, and can cause occupational diseases such as pneumoconiosis. In the presence of ignition sources, suspended coal dust may also trigger explosions. Current dust-control measures mainly include ventilation, dust collectors, and water-spraying systems [1–3]. Ventilation systems generally include forcing ventilation, exhaust ventilation, and hybrid ventilation combining the two.

Considerable efforts have been devoted to improving dust-control performance. Spray-based suppression methods, such as pneumatic atomization and staged spraying, can effectively reduce dust concentrations near cutting zones [4–7]. Airflow-control measures, such as wall-attached air curtains and optimized duct arrangements, can improve airflow organization and enhance dust capture [8–14]. With the development of computational fluid dynamics (CFD), numerical simulations have been widely used to analyze airflow-dust transport at mining workfaces and support the design of ventilation and dust-control strategies [14–19]. Hybrid ventilation combines the advantages of forcing and exhaust ventilation and has been increasingly applied at fully mechanized heading faces [20,21]. Tong et al.[22] proposed an ejector-based staged pneumatic spraying technology and developed a corresponding dust-suppression device.

Despite these advances, two research gaps remain under hybrid ventilation conditions. First, severe dust accumulation is frequently observed at the return-air-side corners when the airflow organization is unfavorable, but the underlying mechanisms and governing factors have not been sufficiently clarified. Second, existing studies have mainly focused on individual ventilation modes or isolated parameters. Systematic investigations of the coupled effects of multiple ventilation parameters, including the exhaust duct inlet angle, exhaust-to-forcing airflow ratio, exhaust duct inlet diameter, and forcing duct outlet diameter, remain limited. Corresponding multi-parameter optimization methods also require further investigation.

In response to these limitations, ANSYS Fluent was used to analyze the effects of key ventilation parameters on dust distribution under a long-forcing, short-exhaust hybrid ventilation system. A dust-control parameter optimization framework integrating CFD simulations, response surface optimization, and scaled-model experimental validation was subsequently established. Following the single-factor analysis, response surface methodology (RSM) was employed to investigate the coupled effects of the exhaust duct inlet angle, exhaust-to-forcing airflow ratio, exhaust duct inlet diameter, and forcing duct outlet diameter. The dust mass concentrations at the lower and upper return-air-side corners were selected as the optimization objectives to determine a favorable combination of ventilation parameters. A 1:4 scaled-model experimental platform was also constructed to validate the numerical results and the optimized configuration. By integrating multi-parameter coupled analysis, response surface optimization, and scaled-model experimental validation, this study provides a systematic approach and an engineering reference for dust control and ventilation parameter optimization under complex mine ventilation conditions.

## 2. Numerical simulation model development

### 2.1. Theoretical basis

Airflow in the roadway of a fully mechanized heading face can be treated as incompressible turbulent flow, while dust particles are transported and dispersed by the turbulent airflow. Based on gas--solid two-phase flow theory and the airflow characteristics in the roadway, the standard k-ε two-equation model was employed for turbulence closure [23]. This model provides good numerical stability and convergence in engineering-scale turbulent-flow simulations and can adequately describe the time-averaged airflow pattern and overall dust-particle transport trends in the roadway.

This study evaluates multiple combinations of ventilation parameters and performs response surface optimization, requiring a balance among computational accuracy, convergence stability, and computational efficiency. The RNG k-ε and realizable k-ε models have certain advantages in predicting strongly swirling flows, streamline curvature, and complex local vortex structures. However, the present study focuses on the variation and optimization trends of dust mass concentrations at the return-air-side corners under different ventilation parameters rather than on detailed transient vortex structures. Therefore, the standard k-ε model was considered suitable for the engineering-scale numerical simulations and parameter optimization conducted in this study.

The governing equations are given as follows:


$$\begin{array}{c} \frac{\partial }{\partial x}\left(\rho \varphi \right)+div\left(\rho \vec{u\varphi }\right)=div\left(\Gamma grad\varphi \right)+S \end{array}$$ (1)

where:

*φ* is the dependent variable;

*u* is the velocity vector;

*Γ* is the diffusion coefficient;

*S* is the source term.

Each control equation can be expressed as follows when *φ* represents the variables:

Continuity equation:


$$\begin{array}{c} \frac{\partial u_{i}}{\partial x_{i}}=0,\frac{\partial {u^{\prime }}_{i}}{\partial x_{i}}=0 \end{array}$$ (2)

Momentum equation:


$$\begin{array}{c} \frac{\partial }{\partial t}\left(\alpha_{i}\rho_{g}u_{i}\right)+\frac{\partial }{\partial x_{i}}\left(\alpha_{i}\rho_{g}u_{j}\right)=F_{sf}+\alpha_{i}\rho_{g}g-\alpha_{f}\frac{\partial p}{\partial x_{i}}+\frac{\partial }{\partial x_{i}}\left(\alpha_{i}\tau_{ij}\right) \end{array}$$ (3)

where:

$T_{\text{ij}}$ is the Reynolds stress term;

$F_{\text{sf}}$ is the force of discrete particles relative to the fluid, N;

p is the distribution function per unit area;

and g is the gravitational acceleration, which is usually taken to be 9.81 m/s².

k equation – turbulent kinetic energy equation:


$$\begin{array}{c} \begin{array}{c} \rho \frac{\partial k}{\partial t}+\rho u_{i}\frac{\partial k}{\partial x_{j}}=\frac{\partial }{\partial x_{j}}\left[\left(\mu +\frac{\mu_{i}}{\Sigma_{k}}\right)\frac{\partial k}{\partial x_{j}}\right]+\mu_{i}\frac{\partial u_{i}}{\partial x_{i}}\left(\frac{\partial u_{i}}{\partial x_{j}}+\frac{\partial u_{j}}{\partial x_{i}}\right)-\rho \epsilon \end{array} \end{array}$$ (4)

ε equation – turbulent energy dissipation rate equation:


$$\begin{array}{c} \rho \frac{\partial \epsilon }{\partial t}+\rho u_{j}\frac{\partial \epsilon }{\partial x_{j}}=\frac{\partial }{\partial x_{j}}\left[\left(\mu +\frac{\mu_{i}}{\Sigma_{k}}\right)\frac{\partial \epsilon }{\partial x_{j}}\right]+\frac{\epsilon c_{\text{1}}}{k}\mu_{i}\frac{\partial u_{i}}{\partial x_{j}}\left(\frac{\partial u_{i}}{\partial x_{j}}+\frac{\partial u_{j}}{\partial x_{i}}\right)-c_{\text{2}}\rho \frac{\epsilon^{\text{2}}}{k} \end{array}$$ (5)

The particle trajectories are modelled in the discrete phase equations by balancing the forces on the discrete particles with the equations:


$$\begin{array}{c} m_{p}\frac{du_{p}}{d_{t}}=m_{p}\frac{u-u_{p}}{\tau_{t}}+m_{p}\frac{g\left(\rho_{g}-\rho \right)}{\rho_{p}}+F \end{array}$$ (6)

where:

$m_{p}$ is the particle mass; *u* is the fluid phase velocity; *F* is the additional force; $m_{p}\frac{\left(u-u_{p}\right)}{\tau_{t}}$ is resistance; $\tau_{t}$ is the particle relaxation time.

### 2.2. Establishment of geometric model

**Fig 1:**
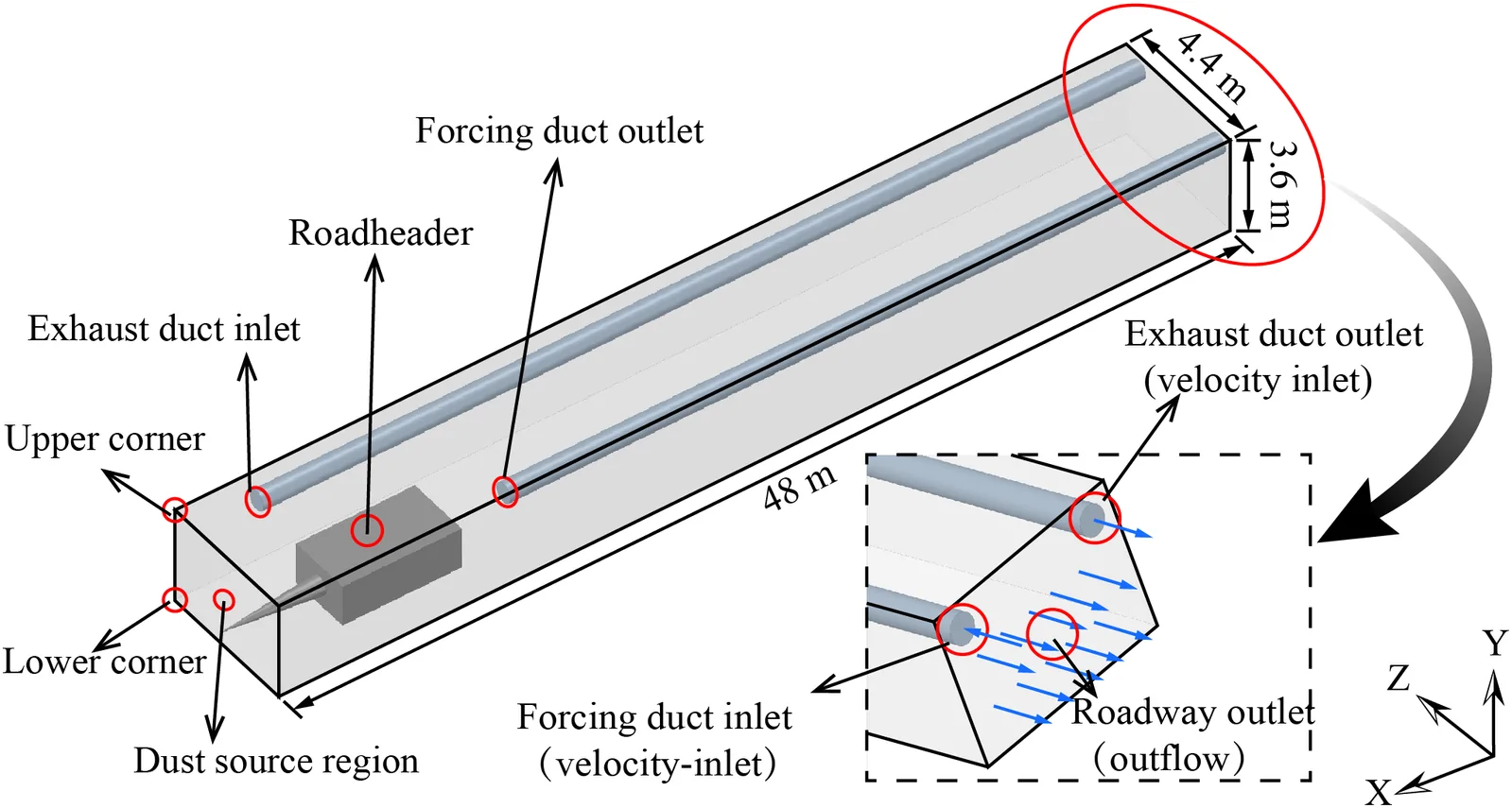
Initial geometry modeling of the mine.

Based on a detailed field investigation conducted at a coal mine in Shanxi Province, a numerical model was developed to investigate dust accumulation in the roadway of a fully mechanized heading face. The actual roadway geometry and equipment arrangement were appropriately simplified using the ANSYS preprocessing module SpaceClaim, and a long-forcing, short-exhaust hybrid ventilation model was established. The roadway dimensions (length × width × height) were 48 × 4.4 × 3.6 m, with a cross-sectional area of approximately 19.895 m^2^. The forcing and exhaust ducts were positioned 0.65 m below the roadway roof and 0.70 m from their respective sidewalls. Both ducts had a diameter of 0.80 m. The forcing duct outlet was located 10 m from the heading face, whereas the exhaust duct inlet was positioned 3 m from the heading face. The roadheader dimensions L × W × H were 9.2 × 2.7 × 1.7 m. The geometric model is shown in Fig 1.

### 2.3. Boundary conditions and numerical settings

During excavation at a fully mechanized heading face, dust is primarily generated when the roadheader cuts the coal and rock masses. Accordingly, a fixed dust source region corresponding to the actual cutting area of the roadheader was defined on the heading face, and dust particles were released using surface injection.

Airflow-dust transport was treated as gas-solid two-phase flow [24]. When the particle volume fraction is below 10%, the gas-solid flow can be regarded as dilute-phase flow, in which particle-particle collisions and agglomeration are relatively weak. Therefore, the Euler-Lagrange method was employed. Air was treated as the continuous phase and solved in an Eulerian framework, whereas dust particles were treated as the discrete phase and tracked in a Lagrangian framework. The particle-size distribution followed the Rosin-Rammler distribution, and the turbulent dispersion of dust particles was modeled using the Discrete Random-Walk Model (DRW). For particle-wall interactions, the trap condition was applied to the roadway floor and the exhaust duct inlet, whereas the reflect condition was applied to the roadway roof, sidewalls, ventilation duct walls, and roadheader surface. These conditions represented particle deposition on the floor, particle collisions with solid walls, and dust removal through the exhaust duct inlet.

The boundary conditions were specified according to the field ventilation parameters listed in Tables 1 and 2. The main boundary conditions were as follows:

**Table 1:** Parameter setting of discrete phase model.

Setting options	Preferences
Phase Coupling	Open
DPM Iteration Interval	20
Injection type	Surface
Particle material	Coal-mv
Mass flow rate (kg·s^−1^)	0.0076
Diameter distribution	Rosin–Rammler
Maximum Diameter (m)	2.6 × 10 ^−4^
Mean Diameter (m)	6.1 × 10 ^− 5^
Minimum Diameter (m)	1.0 × 10 ^− 6^
Particle turbulent dispersion model	Discrete Random-Walk Model
Number of Diameters	10
Spread Parameter	2.87
Discrete phase wall boundary types	Floor: trap; Walls: reflect

**Table 2:** Boundary conditions.

Boundary condition	Preferences	
Forcing duct inlet	Boundary type	Velocity inlet
	Inlet flow rate (m·s^−1^)	8.5
	Turbulence intensity (%)	2.83
	Hydraulic diameter (m)	0.8
Exhaust duct inlet	Boundary type	Velocity inlet
	Inlet flow rate (m·s^−1^)	−1.7, −2.55, −3.4, −4.25, −5.1
	Turbulence intensity (%)	3.17, 3.01, 2.9, 2.82, 2.76
	Hydraulic diameter (m)	0.8
Roadway outlet	Outflow	

Forcing duct inlet: A velocity inlet boundary was specified with an air velocity of 8.5m·s^−1^, a turbulence intensity of 2.83%, and a hydraulic diameter of 0.80 m.

Exhaust duct inlet: A velocity inlet boundary was specified, with the air velocity adjusted according to the exhaust-to-forcing airflow ratio. The prescribed velocity ranged from −1.7 to −5.1 m·s^−1^, where the negative sign indicates the exhaust direction. The turbulence intensity ranged from 2.76 to 3.17, and the hydraulic diameter was 0.80 m.

Roadway outlet: An outflow boundary was specified.

Solid walls: The roadway roof, floor, sidewalls, ventilation duct walls, and roadheader surface were specified as stationary no-slip walls. For the discrete phase, the trap condition was applied to the roadway floor, while the reflect condition was applied to the other solid walls.

The discrete-phase parameters, including the particle mass flow rate, particle-size distribution, and turbulent dispersion model, are listed in Table 1.

The numerical calculations were performed using ANSYS Fluent 2023 R1. Pressure-velocity coupling was handled using the SIMPLE algorithm. The numerical solution was considered converged when all scaled residuals decreased below 10^−5^.

### 2.4. Grid independence analysis

To minimize the influence of mesh resolution on the numerical results, a mesh-independence study was conducted for the computational model. Different meshing strategies were adopted according to the geometric characteristics of the computational domains. Structured meshes were generated for the forcing and exhaust ducts, whereas the roadway and roadheader regions were discretized using unstructured tetrahedral cells. Local mesh refinement was applied near the forcing duct outlet, exhaust duct inlet, roadheader surface, and dust-release surface, where relatively large gradients in airflow velocity and dust mass concentration were expected.

Five mesh schemes with progressively increasing resolutions were generated. The volume-mesh sizes in the roadway region were set to 0.30, 0.26, 0.22, 0.19, and 0.17 m, respectively, whereas those in the ventilation ducts were set to 0.20, 0.16, 0.14, 0.12, and 0.10 m, respectively. For each mesh scheme, the same local face-mesh size was applied to the forcing duct outlet, exhaust duct inlet, roadheader surface, and dust-release surface. The corresponding local mesh sizes were 0.10, 0.08, 0.06, 0.05, and 0.04 m, respectively. These settings produced meshes containing approximately 4.0 × 10^5^, 6.0 × 10^5^, 8.0 × 10^5^, 1.0 × 10^6^, and 1.2 × 10^6^ cells.

To ensure a consistent comparison, simulations for all mesh schemes were performed using identical boundary conditions, solver settings, dust-release parameters, stochastic particle-tracking parameters, and post-processing procedures. The maximum skewness values of all meshes were below 0.79, and the minimum orthogonal quality values were above 0.20, indicating that the mesh quality was acceptable for the numerical calculations.

**Fig 2:**
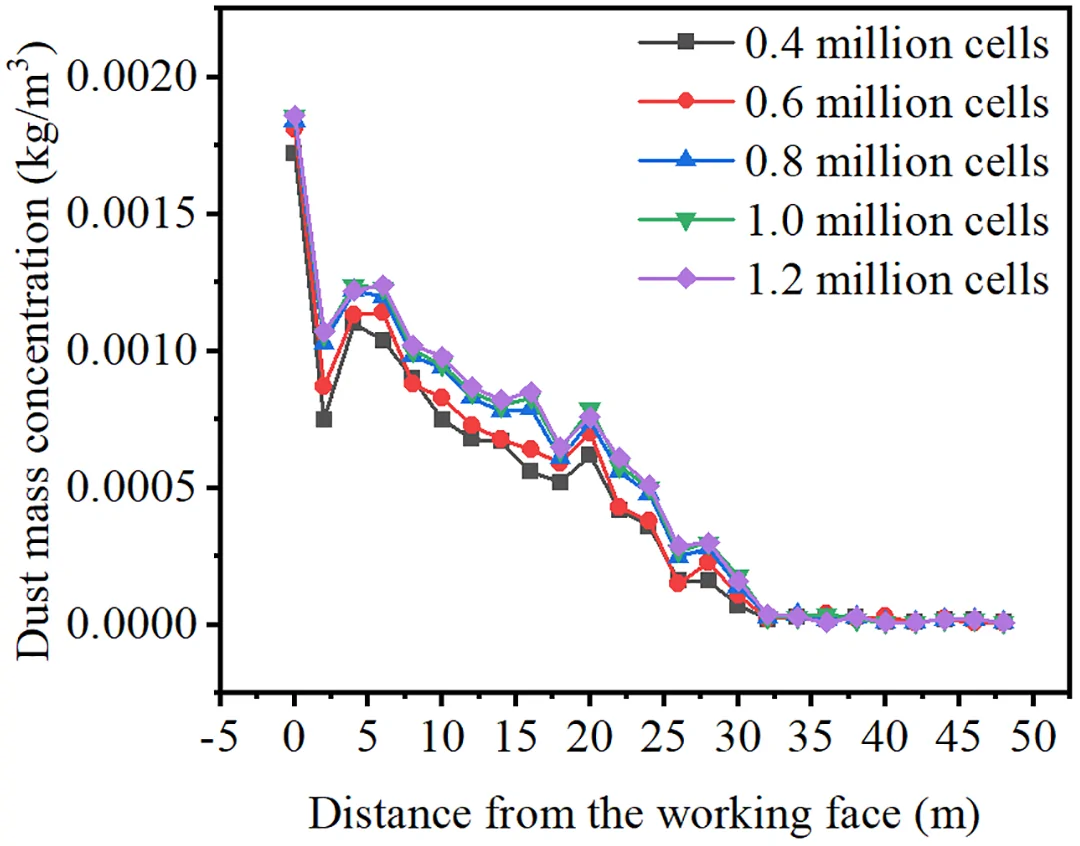
Grid independence test.

The dust mass concentration profile along a longitudinal monitoring line passing through the operator’s position was selected as the evaluation criterion, as shown in Fig 2. The dust mass concentration profiles changed noticeably as the cell count increased from 0.4 million to 0.8 million, indicating that the coarser meshes did not adequately capture dust transport. When the cell count was further increased to 1.0 million and 1.2 million, the peak locations, overall distribution trends, and low-concentration region toward the end of the monitoring line remained nearly unchanged, and the differences among the profiles became substantially smaller.

The average dust mass concentrations along the monitoring line obtained using the 0.8-, 1.0-, and 1.2-million-cell meshes were approximately 5.14 × 10^−4^, 5.30 × 10^−4^, and 5.35 × 10^−4^ kg·m^−3^, respectively. The corresponding maximum values were approximately 1.84 × 10^−3^, 1.86 × 10^−3^, and 1.86 × 10^−3^ kg·m^−3^, respectively. Compared with the results obtained using the 0.8-million-cell mesh, refinement to 1.0 million and 1.2 million cells changed the average dust mass concentration by approximately 3.1% and 4.1%, respectively, whereas the maximum dust mass concentration changed by approximately 1.09% in both cases. These results indicate that further mesh refinement had only a limited effect on the principal numerical results once the cell count reached approximately 0.8 million. The numerical results can therefore be regarded as essentially mesh independent at this resolution. Considering computational accuracy, convergence stability, and computational cost, the mesh containing approximately 0.8 million cells was selected for the subsequent numerical simulations.

### 2.5. Assumptions

Airflow in the roadway of a fully mechanized heading face can be treated as incompressible turbulent flow. When the volume fraction of dust particles is below 10%, the airflow-dust system can be regarded as dilute gas-solid two-phase flow, consistent with the classification commonly adopted in particle-laden flow studies [25].

To simplify the numerical model, the following assumptions were adopted:

(1) The effects of variations in temperature and humidity on airflow and dust-particle transport were neglected. This assumption is commonly adopted in mine ventilation simulations because temperature and humidity in underground ventilation systems are generally considered relatively stable. Their effects on airflow organization and dust transport are also secondary compared with the effects induced by mechanical ventilation [26].
(2) Dust particles were assumed to be spherical and non-cohesive. Their particle-size distribution was described using the Rosin--Rammler model, which has been widely used to characterize coal dust particle-size distributions and simplify complex particle geometries in discrete-phase modeling [27].
(3) Because the volume fraction of dust particles remained below 10%, the gas-solid flow was classified as dilute-phase flow. Under these conditions, interparticle interactions are relatively weak, and particle-particle collisions can be neglected without significantly affecting the overall dust-transport characteristics, as reported in previous studies of particle-laden turbulent flows [28].

These assumptions simplified the numerical model but may have excluded certain secondary effects, such as particle agglomeration and environmental fluctuations. Therefore, the simulation results were primarily used to evaluate the relative variation trends of dust distribution under different ventilation parameters. The absolute numerical values should be interpreted within the scope of the stated assumptions.

## 3. Dust transport patterns and ventilation optimization simulation

### 3.1. Analysis of dust transport patterns

**Fig 3:**
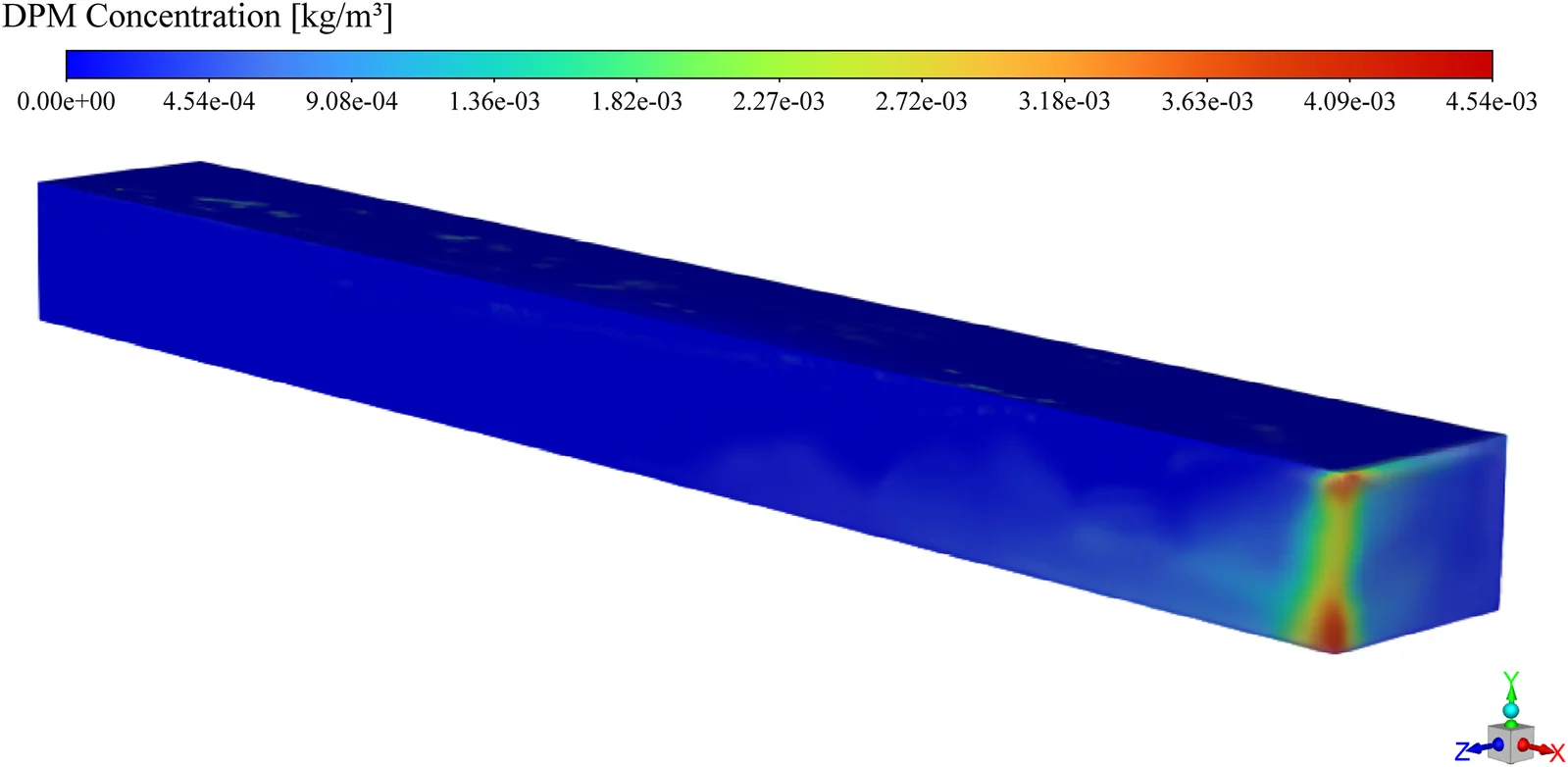
Dust concentration contours on the return-air side under the baseline ventilation condition.

Based on the boundary conditions and numerical settings described in Section 2.3, the dust distribution under the baseline ventilation condition was investigated through numerical simulation. The baseline condition corresponded to an air velocity of −2.55 m·s^−1^ at the exhaust duct inlet, where the negative sign indicates the exhaust direction. ANSYS Fluent was used to obtain the dust mass concentration distribution on the return-air side of the fully mechanized heading face, as shown in Fig 3.

As shown in Fig 3, dust was distributed unevenly across the roadway cross-section. The high-concentration regions were mainly located at the upper and lower return-air-side corners, forming two local dust accumulation zones. The dust mass concentration gradually decreased from the corner regions toward the adjacent main airflow region, indicating relatively weak airflow exchange between the return-air-side corners and the main airflow in the roadway. The highest dust mass concentration occurred at the lower return-air-side corner, reaching 4.52 × 10^−3^ kg·m^−3^, whereas the concentration at the upper return-air-side corner was 3.93 × 10^−3^ kg·m^−3^. The concentration at the lower return-air-side corner was approximately 15.0 higher than that at the upper return-air-side corner.

Because the corner regions are constrained by the roadway walls, momentum from the main airflow cannot be effectively transferred into these regions. Consequently, low-velocity stagnant flow or local recirculation can develop, preventing dust particles from being removed promptly. At the lower return-air-side corner, the near-wall low-velocity effect and gravitational settling jointly increase the residence time of dust particles, resulting in more pronounced dust accumulation. The upper return-air-side corner is also affected by local recirculation, but its dust mass concentration is relatively lower. These concentration gradients and local high-concentration zones indicate that the dust problem on the return-air side is not characterized by a uniform increase in dust mass concentration throughout the roadway but mainly by persistent dust accumulation in the corner regions.

Therefore, ventilation parameter optimization should not only reduce the overall dust mass concentration in the roadway but also specifically mitigate the high-concentration zones at the upper and lower return-air-side corners and enhance airflow exchange between these regions and the main airflow. Adjusting the exhaust duct inlet angle, exhaust-to-forcing airflow ratio, exhaust duct inlet diameter, and forcing duct outlet diameter can modify the effective ranges and momentum balance of the forcing and exhaust airflows, thereby improving local airflow organization and dust removal on the return-air side. This analysis provides a basis for the subsequent single-factor analysis and multi-parameter response surface optimization.

### 3.2. Ventilation System Improvement and Single-Factor Optimization

**Fig 4:**
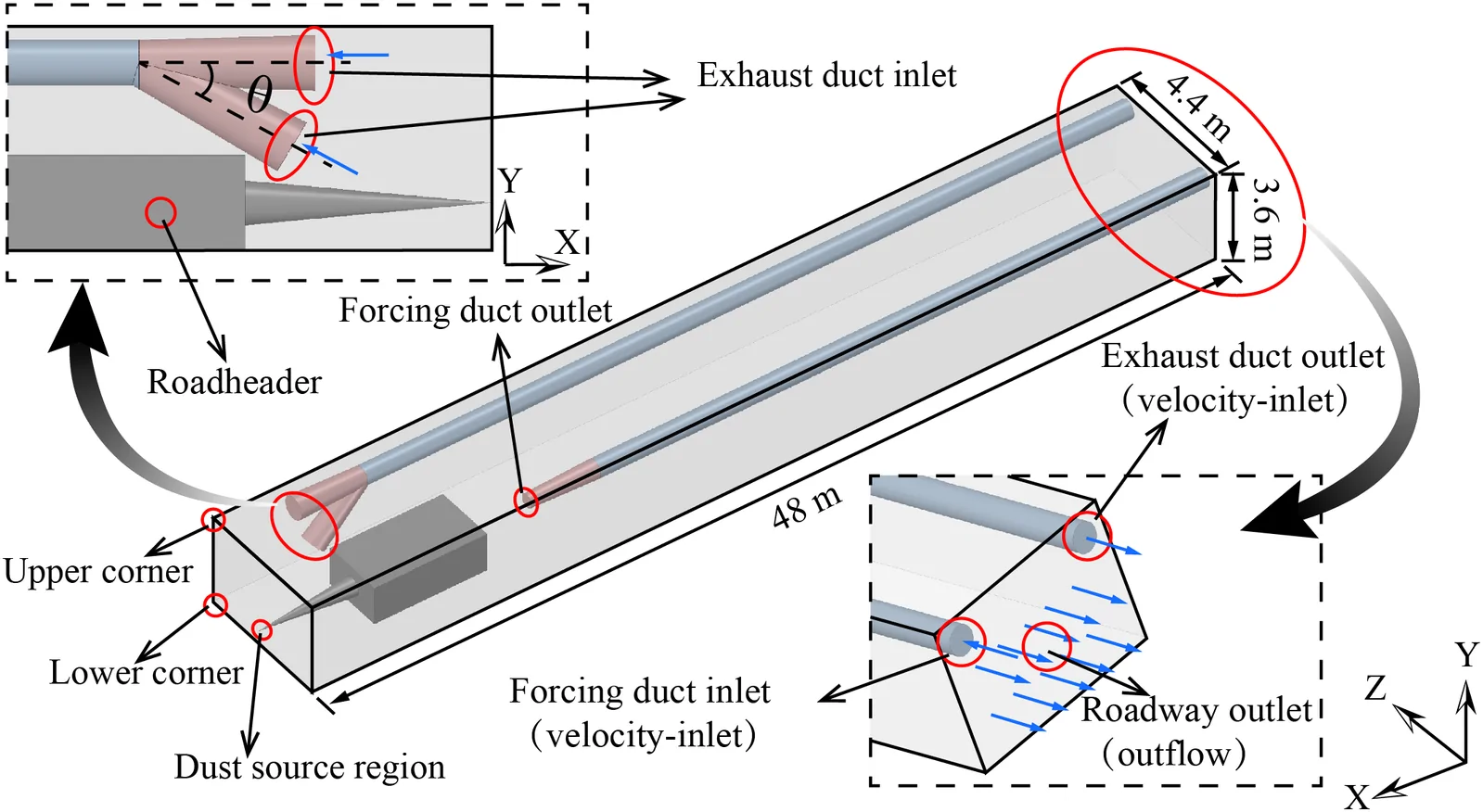
Improved geometry modeling.

Preliminary simulations revealed severe dust accumulation at the return-air-side corners, primarily because the original exhaust duct inlet was positioned too far above the roadway floor to effectively remove dust from the lower part of the roadway. Therefore, additional exhaust inlets were installed on the lower surface of the exhaust duct to improve dust removal near the floor [29]. The parameters of the forcing duct outlet and exhaust duct inlets were subsequently adjusted to improve airflow organization, as shown in Fig 4.

In a long-forcing, short-exhaust hybrid ventilation system, dust accumulation at the return-air-side corners is closely related to the effective range of the exhaust airflow, the balance between the forcing and exhaust airflow rates, and the dust-transport capacity of the forcing-air jet. Based on these factors, the exhaust duct inlet angle (A), exhaust-to-forcing airflow ratio (B), exhaust duct inlet diameter (C), and forcing duct outlet diameter (D) were selected as the main optimization variables. The exhaust duct inlet angle affects the direction and effective range of the exhaust airflow in the corner regions. The exhaust-to-forcing airflow ratio determines the balance between the forcing and exhaust airflow rates. The exhaust duct inlet diameter affects the local exhaust intensity and effective exhaust range, whereas the forcing duct outlet diameter influences the velocity of the forcing-air jet and its capacity to transport dust. These four parameters can be adjusted under field conditions and collectively characterize the effects of the hybrid ventilation system on dust transport and accumulation at the return-air-side corners.

Based on the selected parameters, single-factor analysis was performed to investigate the effects of the exhaust duct inlet angle, exhaust-to-forcing airflow ratio, exhaust duct inlet diameter, and forcing duct outlet diameter on dust distribution. In each single-factor analysis, only one parameter was varied, whereas the other parameters were maintained at the baseline values defined in Section 2.3. The established geometric model was used to simulate dust distribution under different ventilation parameter conditions. The dust mass concentrations at the upper and lower return-air-side corners were selected as the evaluation responses to determine the effect of each parameter on dust accumulation and identify its favorable range.

(1) Effect of the Exhaust Duct Inlet Angle on Dust Accumulation

**Fig 5:**
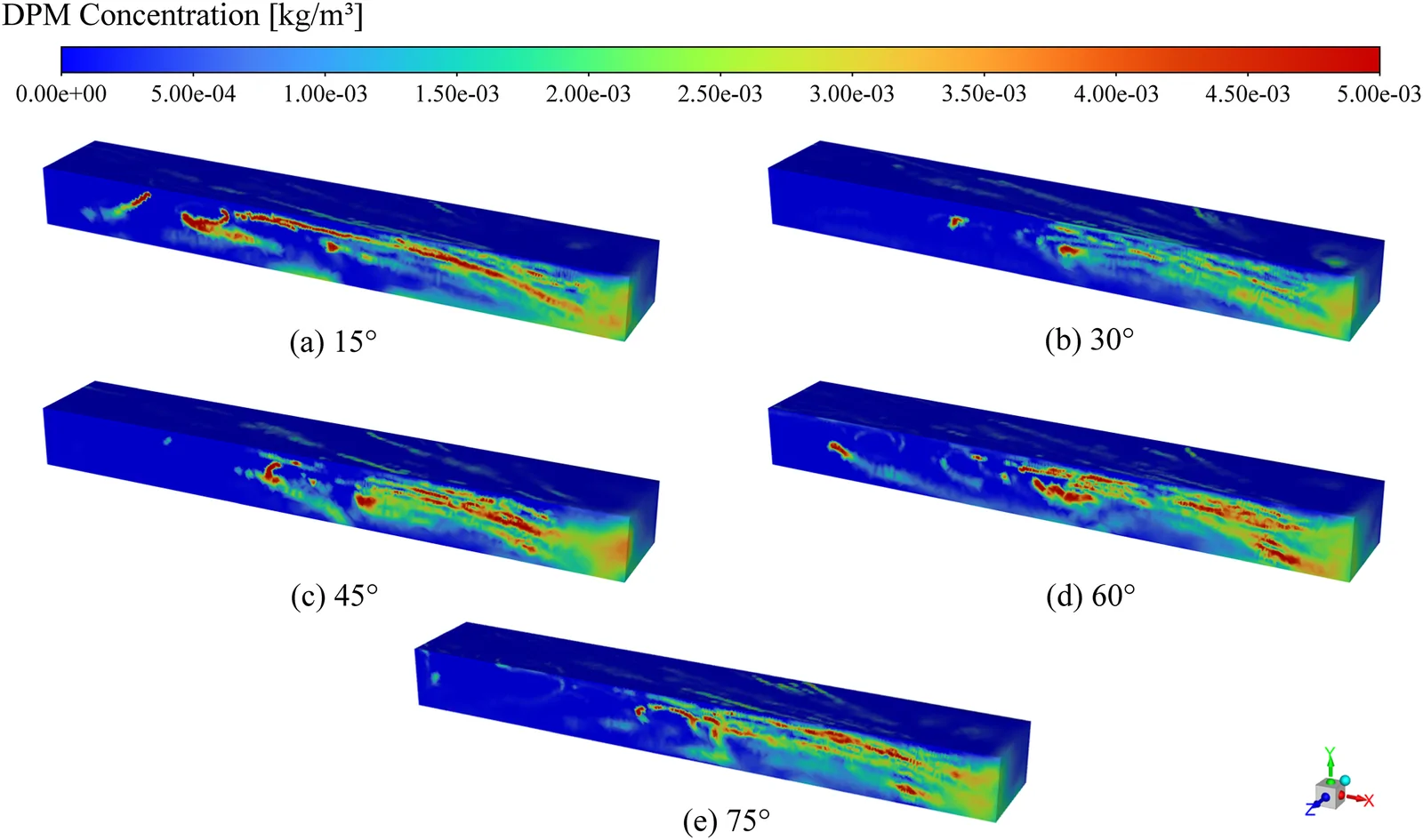
Dust concentration contours under different exhaust duct inlet angles.

**Fig 6:**
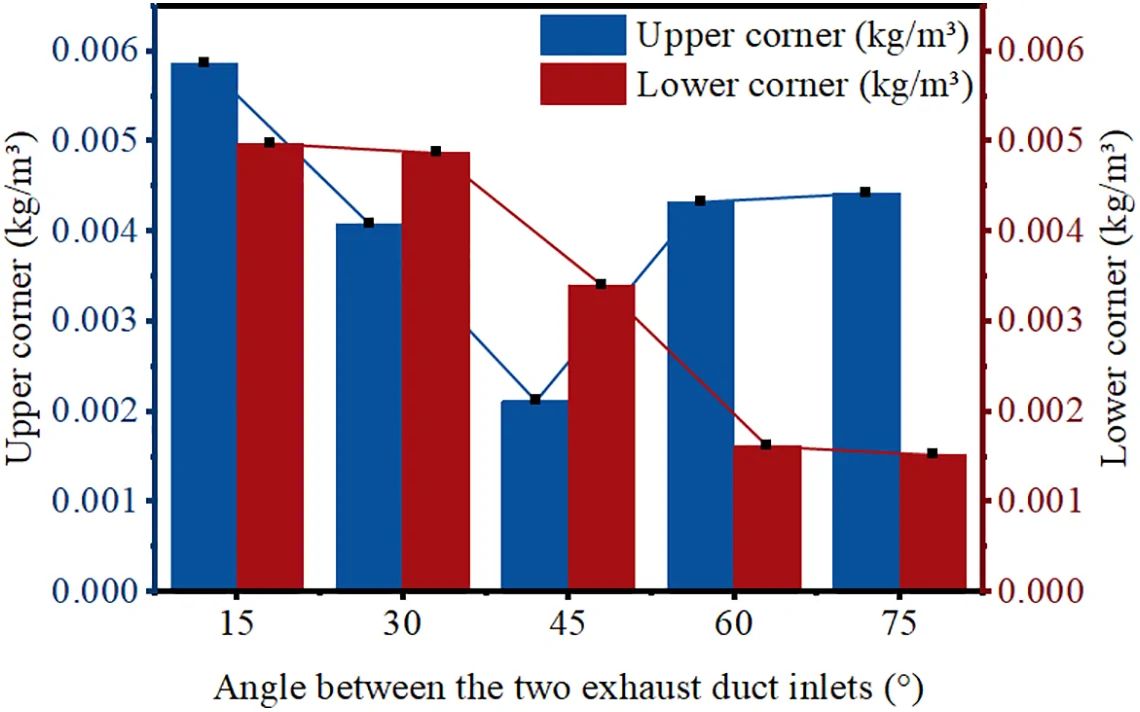
Effect of exhaust duct inlet angle on dust distribution.

To investigate the effect of the exhaust duct inlet angle on dust accumulation, numerical simulations were conducted by varying the included angle between the two exhaust duct inlets from 15° to 75°. The exhaust-to-forcing airflow ratio was maintained at 0.3, while the exhaust duct inlet diameter and forcing duct outlet diameter were both maintained at 0.8 m. The dust mass concentrations at the upper and lower return-air-side corners were monitored to evaluate the effect of the exhaust duct inlet angle on dust distribution, as shown in Figs 5 and 6.

The results indicate that the dust mass concentration at the lower return-air-side corner gradually decreases as the exhaust duct inlet angle increases, with the greatest reduction occurring at 75°. In contrast, the dust mass concentration at the upper return-air-side corner initially decreases and then increases. When the exhaust duct inlet angle increases from 15° to 45°, the dust mass concentrations at both return-air-side corners decrease. As the angle further increases from 45° to 75°, the orientation of one exhaust duct inlet shifts toward the lower return-air-side corner. This change further reduces the dust mass concentration at the lower return-air-side corner but causes a slight increase at the upper return-air-side corner.

Considering the variations in dust mass concentration at both the upper and lower return-air-side corners, an exhaust duct inlet angle ranging from 45° to 75° provides better overall dust-control performance.

(2) Effect of the Exhaust-to-Forcing Airflow Ratio on Dust Accumulation

To investigate the effect of the exhaust-to-forcing airflow ratio on dust accumulation, numerical simulations were performed by varying the ratio from 0.2 to 0.6. The corresponding air velocities at the exhaust duct inlet were −1.70, −2.55, −3.40, −4.25, and −5.10 m·s^−^ 1, where the negative sign indicates the exhaust direction. The air velocity in the forcing duct was maintained constant. During these simulations, the exhaust duct inlet angle was fixed at 45°, while the exhaust duct inlet diameter and forcing duct outlet diameter were both fixed at 0.80 m.

**Fig 7:**
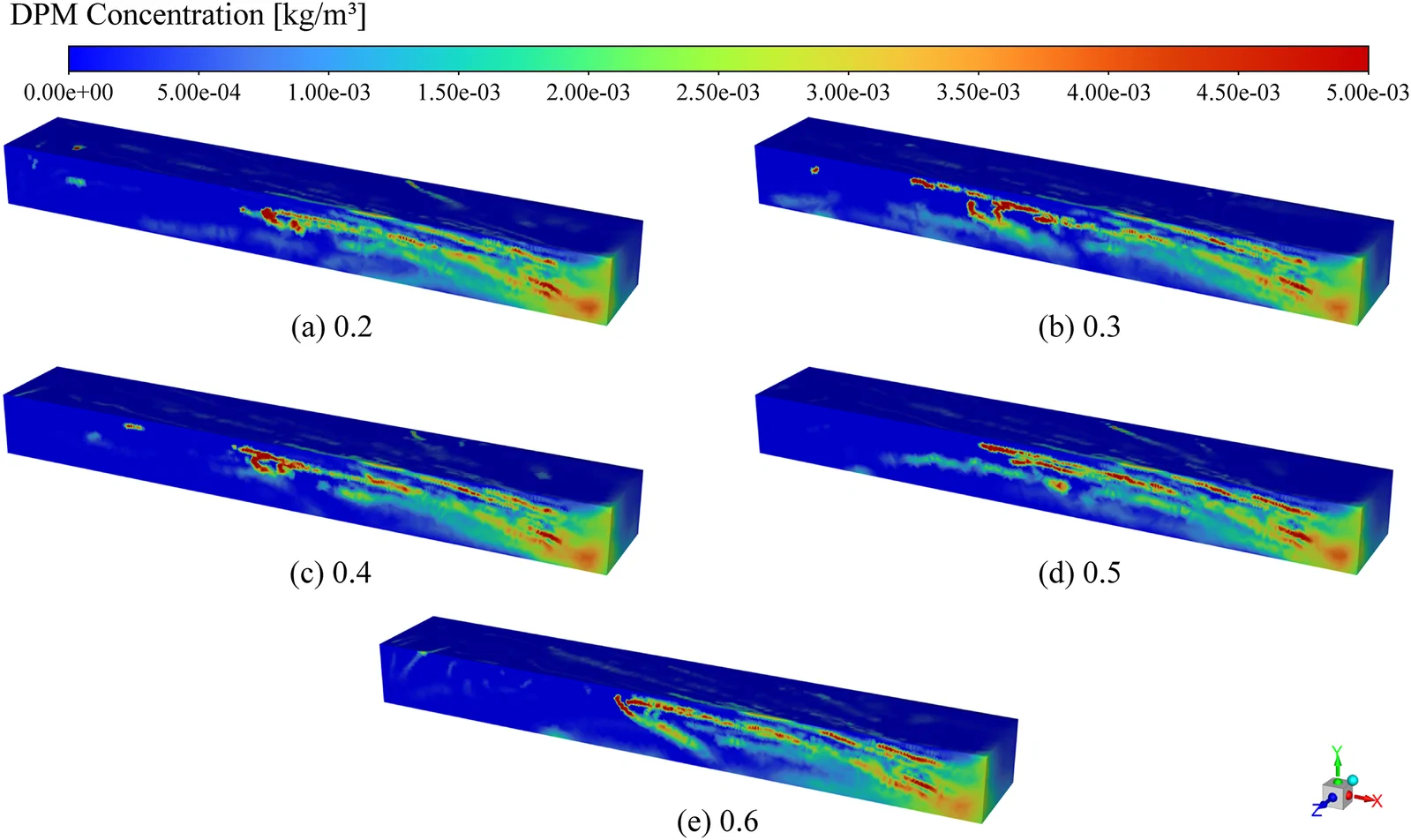
Dust concentration contours under different exhaust-to-forcing airflow ratios.

**Fig 8:**
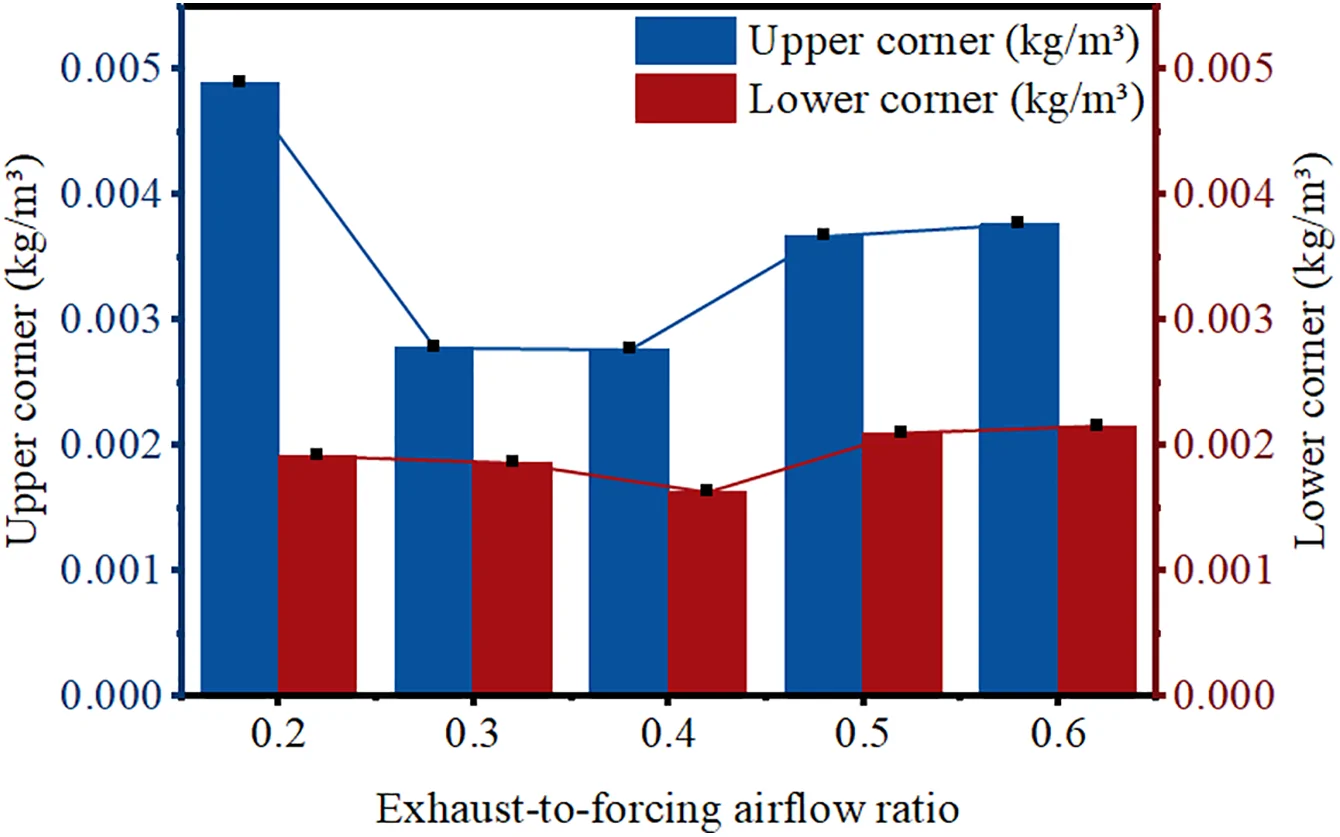
Effect of exhaust-to-forcing airflow ratio on dust concentration.

The simulation results shown in Figs 7 and 8 indicate that the dust mass concentrations at both the upper and lower return-air-side corners initially decrease and then increase as the exhaust-to-forcing airflow ratio increases. The most favorable dust distribution is obtained when the exhaust-to-forcing airflow ratio ranges from 0.3 to 0.5. Within this range, the forcing airflow provides the primary airflow at the heading face, while the exhaust airflow effectively removes dust-laden air from the return-air-side corner regions.

(3) Effect of the Exhaust Duct Inlet Diameter on Dust Accumulation

**Fig 9:**
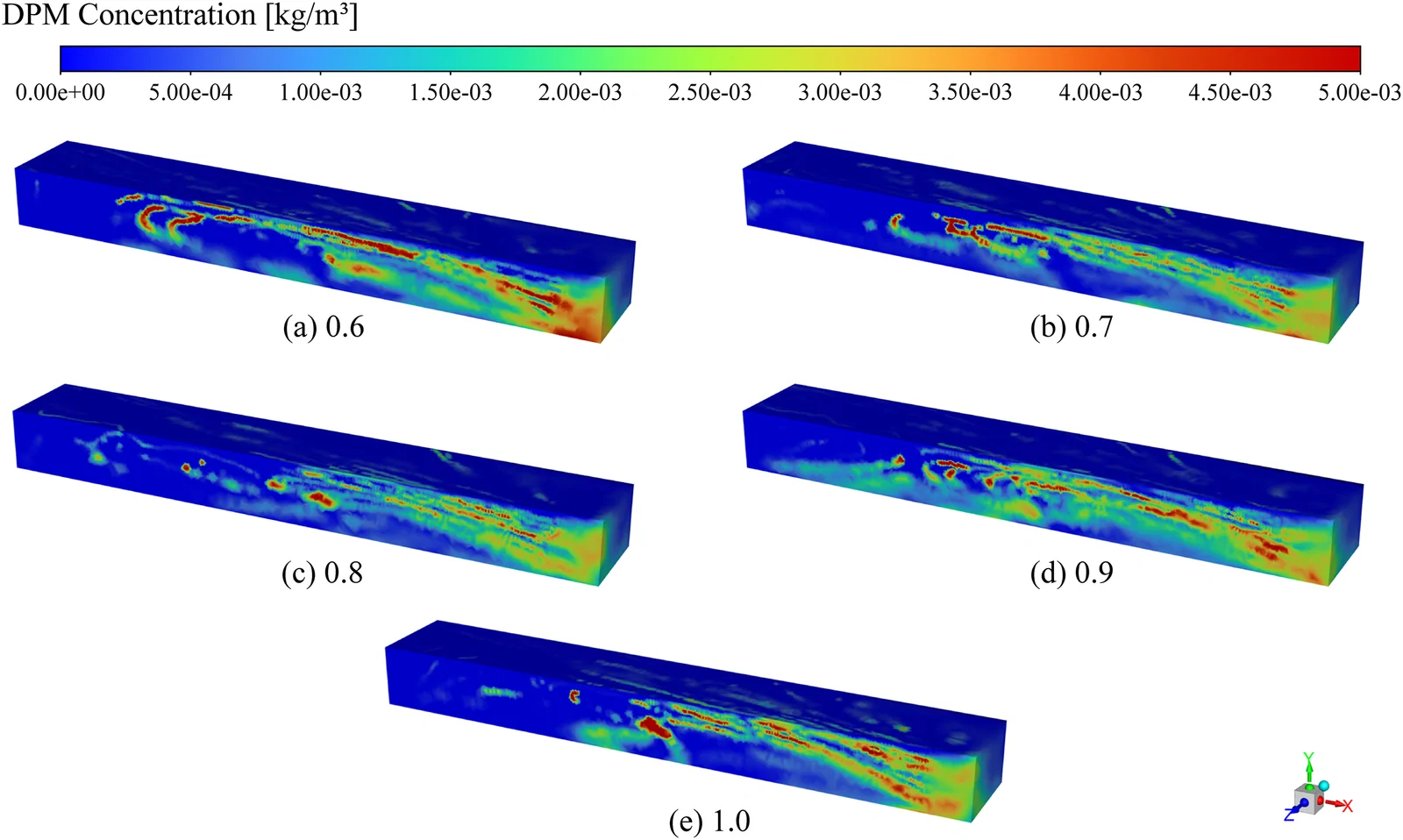
Dust concentration contours under different exhaust duct inlet diameters.

**Fig 10:**
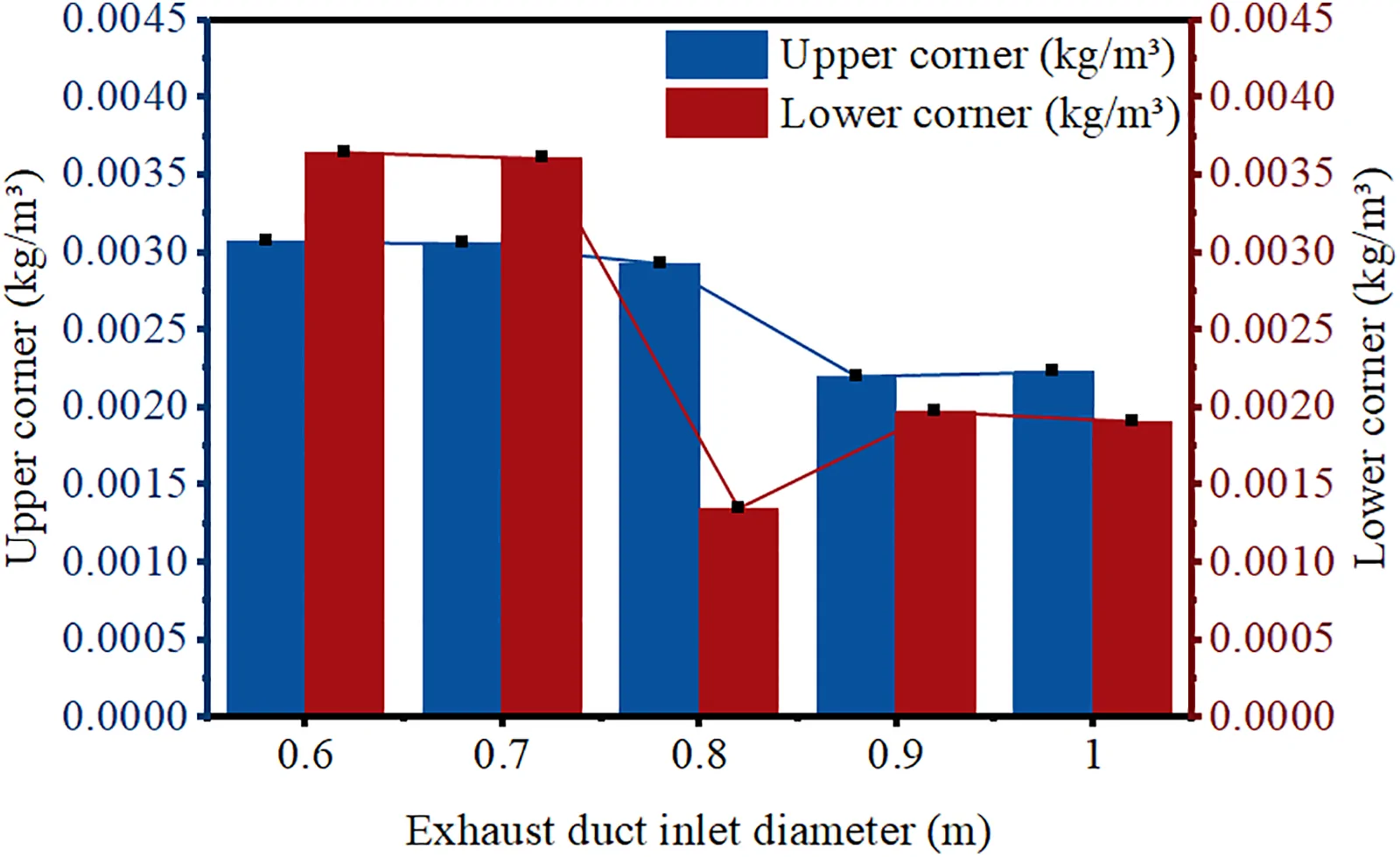
Effect of exhaust duct inlet diameter on dust concentration.

To investigate the effect of the exhaust duct inlet diameter on dust accumulation, numerical simulations were conducted by varying the diameter from 0.6 to 1.0 m. The exhaust duct inlet angle was fixed at 45°, the exhaust-to-forcing airflow ratio was fixed at 0.3, and the forcing duct outlet diameter was fixed at 0.8 m. The dust mass concentrations at the upper and lower return-air-side corners were monitored to evaluate the effect of the exhaust duct inlet diameter on dust distribution, as shown in Figs 9 and 10.

The results indicate that the dust mass concentration at the lower return-air-side corner initially decreases and then increases as the exhaust duct inlet diameter increases, reaching its minimum at a diameter of 0.8 m. In contrast, the dust mass concentration at the upper return-air-side corner decreases continuously with increasing exhaust duct inlet diameter. At a constant exhaust airflow rate, increasing the exhaust duct inlet diameter reduces the local exhaust velocity. However, because the exhaust duct inlet is located relatively close to the heading face, changes in the airflow velocity inside the exhaust duct have a limited effect on the overall airflow structure in the roadway.

Considering the variations in dust mass concentration at both the upper and lower return-air-side corners, an exhaust duct inlet diameter ranging from 0.8 to 1.0 m provides better overall dust-control performance.

(4) Effect of the Forcing Duct Outlet Diameter on Dust Accumulation

**Fig 11:**
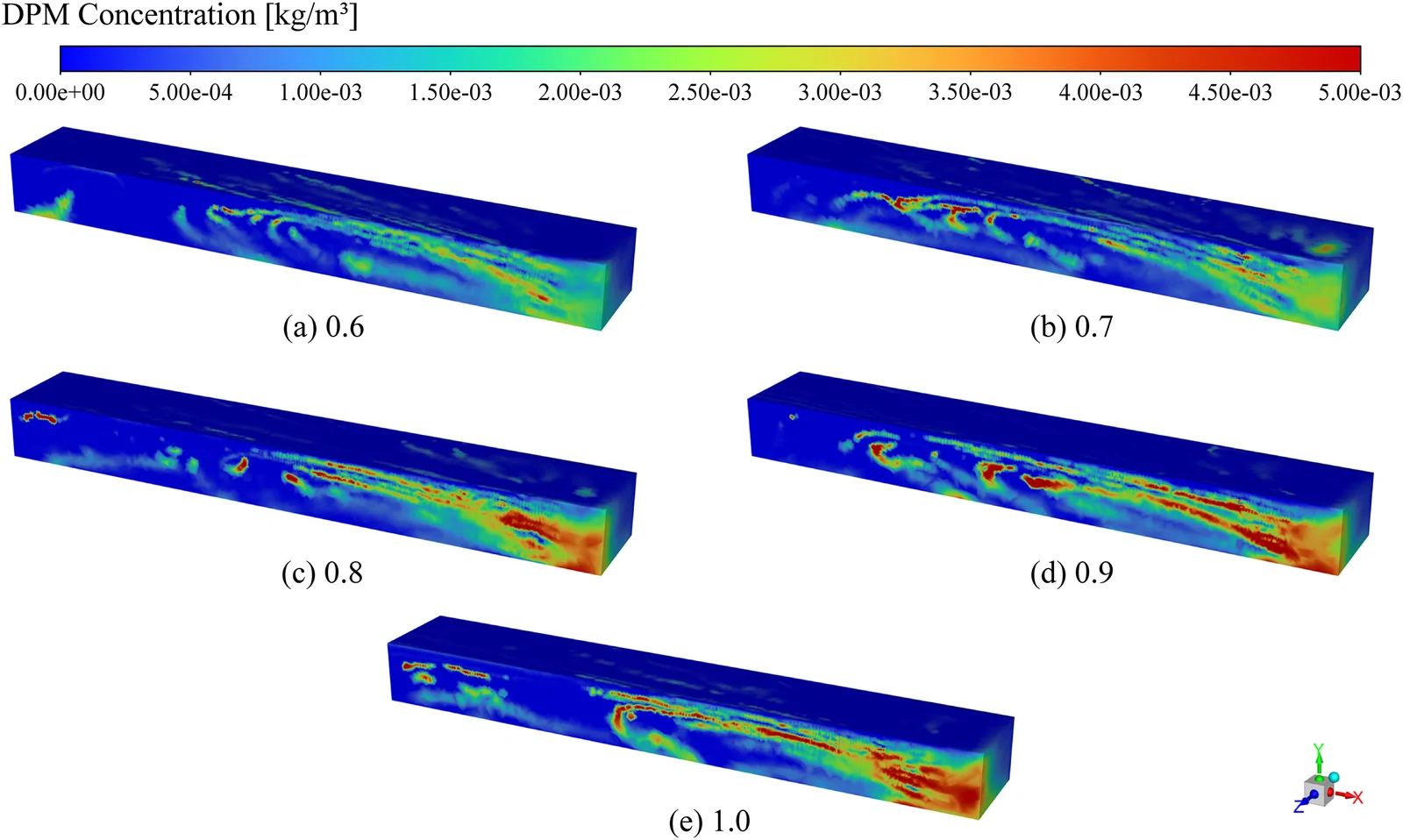
Dust concentration contours under different forcing duct outlet diameters.

**Fig 12:**
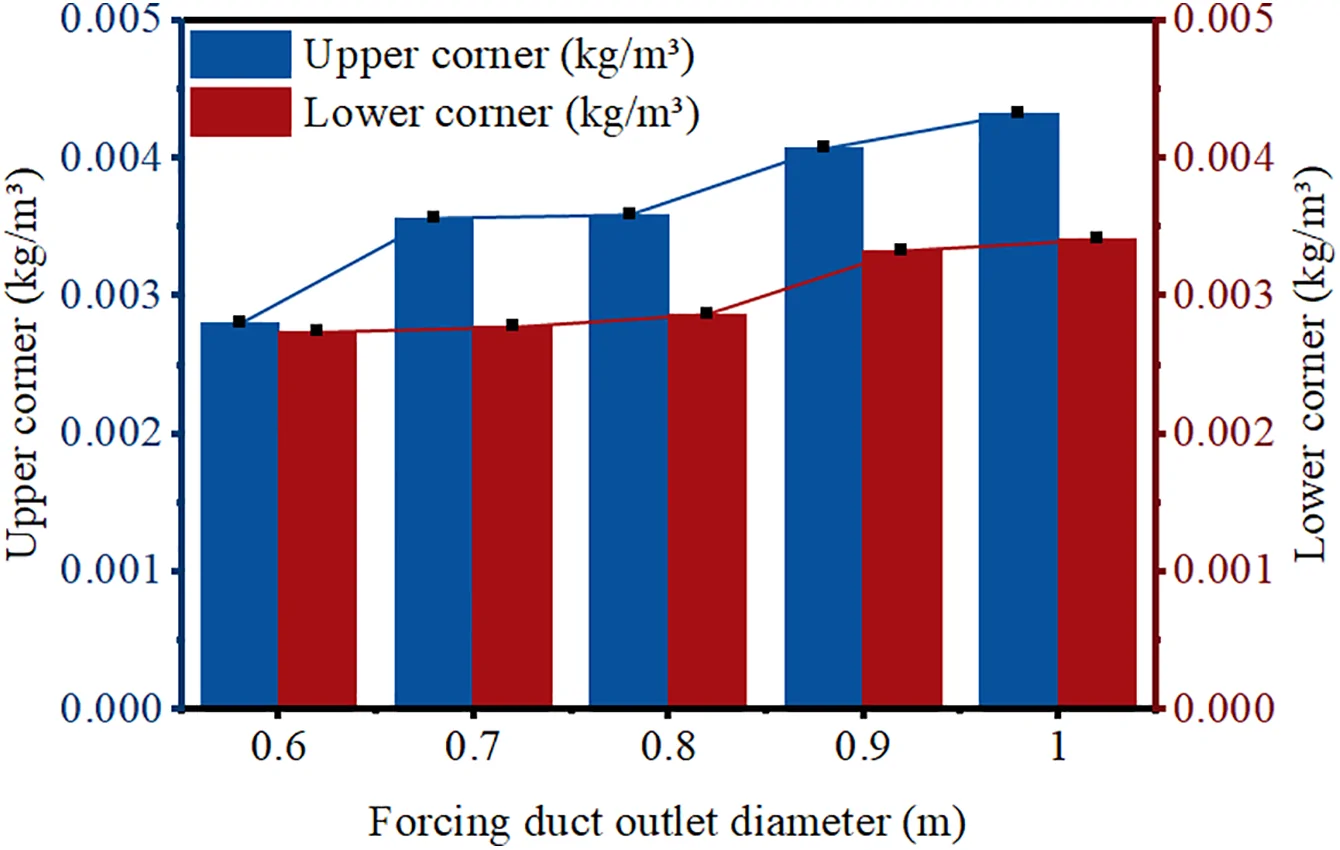
Effect of forcing duct outlet diameter on dust concentration.

To investigate the effect of the forcing duct outlet diameter on dust accumulation, numerical simulations were conducted by varying the diameter from 0.6 to 1.0 m. The exhaust duct inlet angle was fixed at 45°, the exhaust-to-forcing airflow ratio was fixed at 0.3, and the exhaust duct inlet diameter was fixed at 0.8 m. The dust mass concentrations at the upper and lower return-air-side corners were monitored to evaluate the effect of the forcing duct outlet diameter on dust distribution, as shown in Figs 11 and 12.

The results indicate that the dust mass concentrations at the return-air-side corners increase as the forcing duct outlet diameter increases. At a constant forcing airflow rate, a smaller forcing duct outlet diameter produces a higher jet velocity, thereby improving the penetration and dust-transport capacity of the forcing airflow as it reaches the return-air side. This stronger airflow promotes dust removal from the corner regions and reduces local dust accumulation. Therefore, forcing duct outlet diameters ranging from 0.6 to 0.8 m provide better overall dust-control performance.

The single-factor analysis produced the following results. The dust mass concentrations at the return-air-side corners were effectively reduced when the exhaust duct inlet angle ranged from 45° to 75°. The most favorable dust distribution was obtained when the exhaust-to-forcing airflow ratio ranged from 0.3 to 0.5. An exhaust duct inlet diameter ranging from 0.8 to 1.0 m resulted in relatively low dust mass concentrations, particularly at the lower return-air-side corner. A forcing duct outlet diameter ranging from 0.6 to 0.8 m was effective in reducing dust accumulation at both return-air-side corners.

Overall, the single-factor analysis clarified the individual effects of the four ventilation parameters on dust distribution and identified their respective favorable ranges. These results provided a basis for selecting the factor levels used in the subsequent multi-parameter response surface optimization.

### 3.3. Multi-factor response surface optimization

(1) Response Surface Experimental Design

Based on the single-factor analysis, a four-factor, three-level Box-Behnken design (BBD) was developed for the response surface optimization, as shown in Table 3. The design was used to investigate the individual and coupled effects of the exhaust duct inlet angle (A), exhaust-to-forcing airflow ratio (B), exhaust duct inlet diameter (C), and forcing duct outlet diameter (D). A total of 29 numerical test cases were generated to identify a favorable combination of the four ventilation parameters.

**Table 3:** Factor levels for the response surface design.

Level	considerations			
	A(°)	B	C(m)	D(m)
1	45	0.3	0.8	0.6
2	60	0.4	0.9	0.7
3	75	0.5	1.0	0.8

(2) Numerical Simulations and Data Acquisition

Using the established geometric model, numerical simulations were performed for the 29 test cases generated using the Box-Behnken response surface design. Air was treated as the continuous phase and solved using the steady-state standard k-ε turbulence model. Dust particles were treated as the discrete phase, and their trajectories were tracked using the steady-state Discrete Phase Model (DPM). The Discrete Random-Walk Model (DRW) was enabled to account for the effects of turbulent fluctuations on dust dispersion. Random sampling was used to determine the fluctuating velocity experienced by each particle and the corresponding eddy interaction time. The same mesh, boundary conditions, dust-release parameters, solver settings, convergence criteria, and post-processing procedures were used for all test cases. Only the ventilation parameters specified by the response surface design were varied.

After the specified convergence criteria were satisfied, the dust mass concentrations at the lower and upper return-air-side corners were extracted from ANSYS Fluent and denoted as *S*_1_ and *S*_2_, respectively. The results are presented in Table 4. Here, *S*_1_ represents the dust mass concentration at the lower return-air-side corner, whereas *S*_2_ represents that at the upper return-air-side corner; both are expressed in kg·m^−3^. The four ventilation parameters are the exhaust duct inlet angle (A, °), exhaust-to-forcing airflow ratio (B), exhaust duct inlet diameter (C, m), and forcing duct outlet diameter (D, m).

**Table 4:** Response surface simulation results.

design	A/ (°)	B	C/ (m)	D/ (m)	S_1_/ (kg/m³)	S_2_/ (kg/m³)	design	A/ (°)	B	C/ (m)	D/ (m)	S_1_/ (kg/m³)	S_2_/ (kg/m³)
1	75	0.4	0.9	0.6	1.72·10 ^− 3^	1.51·10 ^− 3^	16	45	0.4	0.9	0.6	4.38·10 ^− 4^	3.08·10 ^− 3^
2	75	0.5	0.9	0.7	1.62·10 ^− 3^	2.60·10 ^− 3^	17	60	0.4	1.0	0.8	1.85·10 ^− 3^	1.65·10 ^− 3^
3	60	0.5	0.8	0.7	2.67·10 ^− 3^	2.37·10 ^− 3^	18	60	0.3	0.9	0.8	2.20·10 ^− 3^	1.56·10 ^− 3^
4	60	0.4	0.9	0.7	1.63·10 ^− 3^	2.67·10 ^− 3^	19	75	0.4	1.0	0.7	2.29·10 ^− 3^	2.58·10 ^− 3^
5	60	0.4	0.9	0.7	3.09·10 ^− 3^	2.49·10 ^− 3^	20	60	0.4	0.8	0.8	1.88·10 ^− 3^	2.96·10 ^− 3^
6	75	0.3	0.9	0.7	1.81·10 ^− 3^	1.52·10 ^− 3^	21	60	0.3	1.0	0.7	1.86·10 ^− 3^	2.26·10 ^− 3^
7	60	0.3	0.9	0.6	1.14·10 ^− 3^	1.93·10 ^− 3^	22	60	0.5	0.9	0.6	1.06·10 ^− 3^	2.13·10 ^− 3^
8	45	0.4	0.9	0.8	3.12·10 ^− 3^	3.52·10 ^− 3^	23	60	0.5	1.0	0.7	2.20·10 ^− 3^	2.45·10 ^− 3^
9	75	0.4	0.9	0.8	2.56·10 ^− 3^	1.95·10 ^− 3^	24	45	0.5	0.9	0.7	1.49·10 ^− 3^	2.92·10 ^− 3^
10	75	0.4	0.8	0.7	2.73·10 ^− 3^	2.07·10 ^− 3^	25	60	0.3	0.8	0.7	1.04·10 ^− 3^	2.45·10 ^− 3^
11	45	0.3	0.9	0.7	1.30·10 ^− 3^	3.13·10 ^− 3^	26	60	0.4	0.9	0.7	1.95·10 ^− 3^	2.06·10 ^− 3^
12	60	0.4	1.0	0.6	1.16·10 ^− 3^	1.93·10 ^− 3^	27	60	0.5	0.9	0.8	1.73·10 ^− 3^	3.05·10 ^− 3^
13	60	0.4	0.9	0.7	1.64·10 ^− 3^	2.67·10 ^− 3^	28	60	0.4	0.9	0.7	2.16·10 ^− 3^	2.69·10 ^− 3^
14	45	0.4	1.0	0.7	1.95·10 ^− 3^	2.15·10 ^− 3^	29	60	0.4	0.8	0.6	1.89·10 ^− 3^	2.95·10 ^− 3^
15	45	0.4	0.8	0.7	2.68·10 ^− 3^	2.00·10 ^− 3^							

Designs 4, 5, 13, 26, and 28 in Table 4 are replicated center-point runs. These designs have identical values of A, B, C, and D and identical continuous-phase calculation conditions. However, each center-point run corresponds to an independent steady-state stochastic particle-tracking calculation. Because the steady airflow field is deterministic, the differences among the replicated center-point results are not caused by physical fluctuations in the airflow field. Instead, they mainly arise from numerical sampling variation associated with a finite number of stochastic particle trajectories. All replicated center-point results were included in the statistical analysis to evaluate the repeatability of the numerical calculations, estimate the pure error of the response surface models, and provide data for the subsequent regression analysis and analysis of variance (ANOVA).

Response surface analysis of the *S*_1_ simulation results was performed using Design-Expert software. A quadratic regression model was developed to describe the relationship between the dust mass concentration at the lower return-air-side corner (*S*_1_) and the four ventilation parameters A, B, C, and D [30], as expressed in Eq. 7. The statistical significance and adequacy of the regression model were evaluated using analysis of variance (ANOVA), and the results are presented in Table 5.

**Table 5:** ANOVA analysis of the response surface model for dust concentration at the lower corner.

Source	Sum of Squares	Degree of Freedom	Mean Square	F-value	p-value
Model	3.61 × 10^6^	4	9.02 × 10^5^	2.85	0.046
A	2.61 × 10^5^	1	2.61 × 10^5^	0.824	0.373
B	1.71 × 10^5^	1	1.71 × 10^5^	0.540	0.469
C	2.09 × 10^5^	1	2.09 × 10^5^	0.660	0.425
D	2.97 × 10^6^	1	2.97 × 10^6^	9.36	0.005
Residual	7.61 × 10^6^	24	3.17 × 10^5^		
Lack of Fit	6.16 × 10^6^	20	3.08 × 10^5^	0.850	0.649
Pure Error	1.45 × 10^6^	4	3.63 × 10^5^		
Cor Total	1.12 × 10^7^	28			


$$\begin{array}{c} S_{\text{1}}=1897.10+147.49A+119.47B-131.97C+497.22D \end{array}$$ (7)

As shown in Table 5, the response surface model for *S*_1_ was statistically significant (F = 2.85, p = 0.046), whereas the lack-of-fit term was not significant (F = 0.85, p = 0.649). In the analysis of variance, terms with p < 0.05 were considered statistically significant. These results indicate that the regression model adequately represents the simulation data and can be used to interpret the response trends within the investigated parameter ranges.

Based on the absolute values of the coded regression coefficients, the effects of the four ventilation parameters on the dust mass concentration at the lower return-air-side corner (*S*_1_) followed the order *D* > *A* > *C* > *B*. This result indicates that the forcing duct outlet diameter (*D*) had the greatest effect on *S*_1_, whereas the exhaust-to-forcing airflow ratio (*B*) had the smallest effect. With the minimization of *S*_1_ as the optimization objective, numerical optimization using Design-Expert produced a favorable parameter combination of A = 45.099°, B = 0.3, C = 1.0 m, and D = 0.6 m.

Similarly, response surface analysis was performed for the dust mass concentration at the upper return-air-side corner (*S*_2_). The corresponding quadratic regression model is presented in Eq. 8, and the analysis-of-variance results for the *S*_2_ response surface model are summarized in Table 6.

**Table 6:** ANOVA analysis of the response surface model of dust concentration at the upper corner.

Source	Sum of Squares	Degree of Freedom	Mean Square	F-value	p-value
Model	2.696 × 10^6^	4	6.74 × 10^5^	3.11	0.034
A	1.730 × 10^6^	1	1.73 × 10^6^	7.98	0.009
B	5.877 × 10^5^	1	5.88 × 10^5^	2.71	0.113
C	2.618 × 10^5^	1	2.62 × 10^5^	1.21	0.283
D	1.159 × 10^5^	1	1.16 × 10^5^	0.534	0.472
Residual	5.207 × 10^6^	24	2.17 × 10^5^		
Lack of Fit	4.912 × 10^6^	20	2.46 × 10^5^	3.33	0.126
Pure Error	2.952 × 10^5^	4	7.38 × 10^4^		
Cor Total	7.902 × 10^6^	28			


$$\begin{array}{c} S_{\text{2}}=2392.95-397.75A+221.30B-147.70C+98.28D \end{array}$$ (8)

As shown in Table 6, the response surface model for *S*_2_ was statistically significant at the 95% confidence level, with an F-value of 3.11 and a corresponding p-value of 0.034. In contrast, the lack-of-fit term was not statistically significant, with an F-value of 3.33 and a p-value of 0.126. In the analysis of variance, terms with p < 0.05 were considered statistically significant. These results indicate that the regression model adequately represents the simulation data and is suitable for interpreting the response trends within the investigated parameter ranges.

Based on the absolute values of the coded regression coefficients, the effects of the four ventilation parameters on the dust mass concentration at the upper return-air-side corner (*S*_2_) followed the order *A* > *B* > *C* > *D*. The exhaust duct inlet angle (*A*) had the greatest effect on *S*_2_, whereas the forcing duct outlet diameter (*D*) had the smallest effect.

With the minimization of *S*_2_ as the optimization objective, numerical optimization was performed using Design-Expert. The resulting favorable parameter combination consisted of an exhaust duct inlet angle of *A* = 74.529°, an exhaust-to-forcing airflow ratio of *B* = 0.309, an exhaust duct inlet diameter of *C* = 0.999 m, and a forcing duct outlet diameter of *D* = 0.757 m.

Considering the dust mass concentrations at both the upper and lower return-air-side corners, a dual-objective optimization model was established to simultaneously minimize *S*_1_ and *S*_2_. The optimization problem was formulated as follows:


$$\begin{array}{c} \left\{\begin{array}{c} @c@minF\left(\xi \right)=min\left(S_{\text{1}},S_{\text{2}}\right)\\ \xi_{imin}\leq \xi_{i}\leq \xi_{imax}\left(i=1,2,3,4\right) \end{array}\right\} \end{array}$$ (9)

where $\xi =\left(\xi_{\text{1}},\xi_{\text{2}},\xi_{\text{3}},\xi_{\text{4}}\right)$ denotes the vector of optimization variables corresponding to the exhaust duct inlet angle (*A*), exhaust-to-forcing airflow ratio (*B*), exhaust duct inlet diameter (*C*), and forcing duct outlet diameter (*D*), respectively. The terms $\xi_{imin}$ and $\xi_{imax}$ represent the lower and upper bounds of the *i*-th optimization variable, respectively.

Based on the established regression models for *S*_1_ and *S*_2_, numerical multi-objective optimization was performed using Design-Expert to obtain a compromise solution that simultaneously minimized the dust mass concentrations at the lower and upper return-air-side corners. The optimal solution consisted of an exhaust duct inlet angle of 74.999°, an exhaust-to-forcing airflow ratio of 0.3, an exhaust duct inlet diameter of 1.0 m, and a forcing duct outlet diameter of 0.6 m. Considering practical engineering implementation and operational convenience, the exhaust duct inlet angle was rounded to 75°. Therefore, the final optimized parameter combination was *A* = 75°, *B* = 0.3, *C* = 1.0 m, and *D* = 0.6 m. Under this optimized ventilation scheme, the predicted dust mass concentrations at the lower and upper return-air-side corners were 1.938 × 10^−3^ kg·m^−3^ and 2.598 × 10^−3^ kg·m^−3^, respectively.

### 3.4. Optimal solution and simulation verification

Based on the optimal parameter combination obtained through response surface optimization, namely an exhaust duct inlet angle of 75°, an exhaust-to-forcing airflow ratio of 0.3, an exhaust duct inlet diameter of 1.0 m, and a forcing duct outlet diameter of 0.6 m, an additional numerical simulation was performed to evaluate the dust distribution at the fully mechanized heading face.

**Fig 13:**
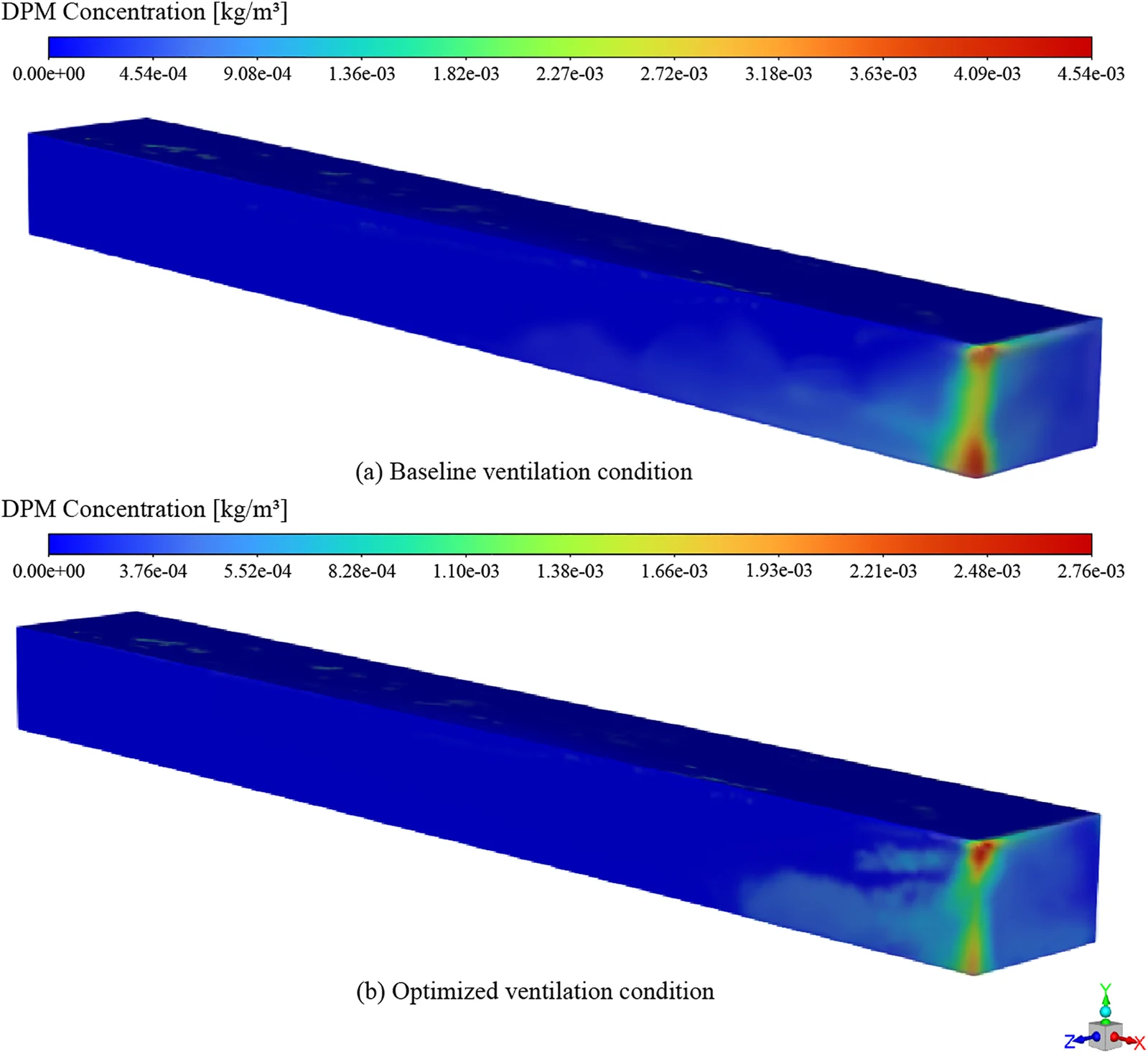
Dust concentration distribution under baseline and optimized ventilation conditions.

The simulation results show that dust accumulation at the return-air-side corners was substantially reduced under the optimized ventilation conditions. Compared with the baseline ventilation scheme, the dust mass concentration decreased by approximately 58% at the lower return-air-side corner (*S*_1_) and 34% at the upper return-air-side corner (*S*_2_), as shown in Fig 13.

The response surface models predicted dust mass concentrations of 1.938 × 10^−3^ kg·m^−3^ at *S*_1_ and 2.598 × 10^−3^ kg·m^−3^ at *S*_2_ under the optimized ventilation conditions. The verification simulation performed using the same optimized parameters produced corresponding values of 1.94 × 10^−3^ kg·m^−3^ and 2.59 × 10^−3^ kg·m^−3^, respectively. The close agreement between the model predictions and verification simulation results supports the predictive accuracy of the response surface models within the investigated parameter ranges.

The reduction in dust mass concentration was mainly attributed to the improved airflow organization produced by the optimized ventilation parameters. Increasing the exhaust duct inlet angle expanded the effective range of the exhaust airflow, thereby facilitating the removal of dust accumulated near the roadway floor. Reducing the forcing duct outlet diameter increased the forcing-air jet velocity and enhanced the capacity of the airflow to dilute and transport dust particles. In addition, optimization of the exhaust duct inlet diameter provided a more suitable balance between the effective exhaust range and the local exhaust velocity.

Overall, the numerical simulation results confirm that the proposed ventilation parameter combination can effectively reduce dust accumulation at the return-air-side corners. The close agreement between the response surface predictions and verification simulation results further indicates that the response surface models provide reasonable estimates of the dust mass concentrations under the optimized conditions. To further evaluate the applicability of the proposed scheme under engineering conditions, scaled-model experiments were subsequently conducted.

## 4. Experimental validation

### 4.1. Experimental platform and test plan

To validate the numerical simulation results and assess the reliability of the optimized ventilation scheme, a 1:4 scaled-model experimental platform representing a fully mechanized heading face was constructed. The experimental roadway was 12 m long, 1.1 m wide, and 0.9 m high, corresponding to one-quarter of the principal geometric dimensions of the prototype roadway. The main structure consisted of a 0.003 m-thick steel frame, and 0.004 m-thick glass panels were installed along both sidewalls to permit observation of airflow organization and dust transport during the experiments, as shown in Fig 13(a).

The experimental platform was designed according to similarity principles so that the scaled model could reproduce the principal airflow organization and dust-transport characteristics of the prototype roadway. To ensure geometric similarity, the roadway length, width, and height, ventilation duct diameters, duct positions, and measurement-point locations were all scaled at a ratio of 1:4. For kinematic similarity, the forcing and exhaust air velocities were adjusted so that the airflow conditions in the scaled model corresponded to those in the prototype, while the principal ventilation regions remained within the turbulent-flow regime. For dynamic similarity, the Reynolds number was adopted as the primary reference parameter for evaluating airflow similarity and was calculated as


$$\begin{array}{c} Re=\frac{\rho uD}{\mu } \end{array}$$ (10)

Where *ρ* is the air density, *u* is the characteristic air velocity, *D* is the characteristic length, and *μ* is the dynamic viscosity of air. The Reynolds numbers of both the scaled experimental model and the prototype roadway were within the turbulent-flow regime, indicating that their dominant airflow characteristics were comparable. For dust-particle transport, the Stokes number was adopted as a reference parameter to characterize the relationship between particle inertia and the ability of the airflow to transport particles. The dust-release location, release method, and measurement-point arrangement corresponded to those used in the numerical model. Therefore, the scaled-model experimental platform could be used to evaluate changes in dust mass concentration under different ventilation parameters and verify the effectiveness of the optimized scheme.

The experimental system mainly consisted of the experimental roadway, dust generator, forcing-air unit, exhaust-air unit, ventilation ducts, and dust mass concentration sensors. A TDA-4B coal dust generator was used to provide a stable and continuous dust source. The dust-release rate was controlled primarily by adjusting the conveyor-belt speed and the negative pressure of the generator, as shown in Figs 13(b1) and 13(b4). After each experiment began, dust was continuously released from the generator and transported through the roadway by the ventilation airflow.

The ventilation system consisted of a forcing duct, an exhaust duct, and portable axial ventilation fans. The forcing duct had a diameter of 0.20 m and was positioned 0.16 m below the roadway roof and 0.18 m from the adjacent sidewall. The forcing duct outlet was located 2.50 m from the dust-release point, whereas its inlet was connected to a 16-inch portable axial ventilation fan to provide the forcing airflow. The exhaust duct inlet was positioned 1.25 m from the dust-release point, while its rear end was connected to a 12-inch portable axial ventilation fan. The air velocity in the exhaust duct and the exhaust-to-forcing airflow ratio were controlled by adjusting the operating condition of the exhaust fan.

**Fig 14:**
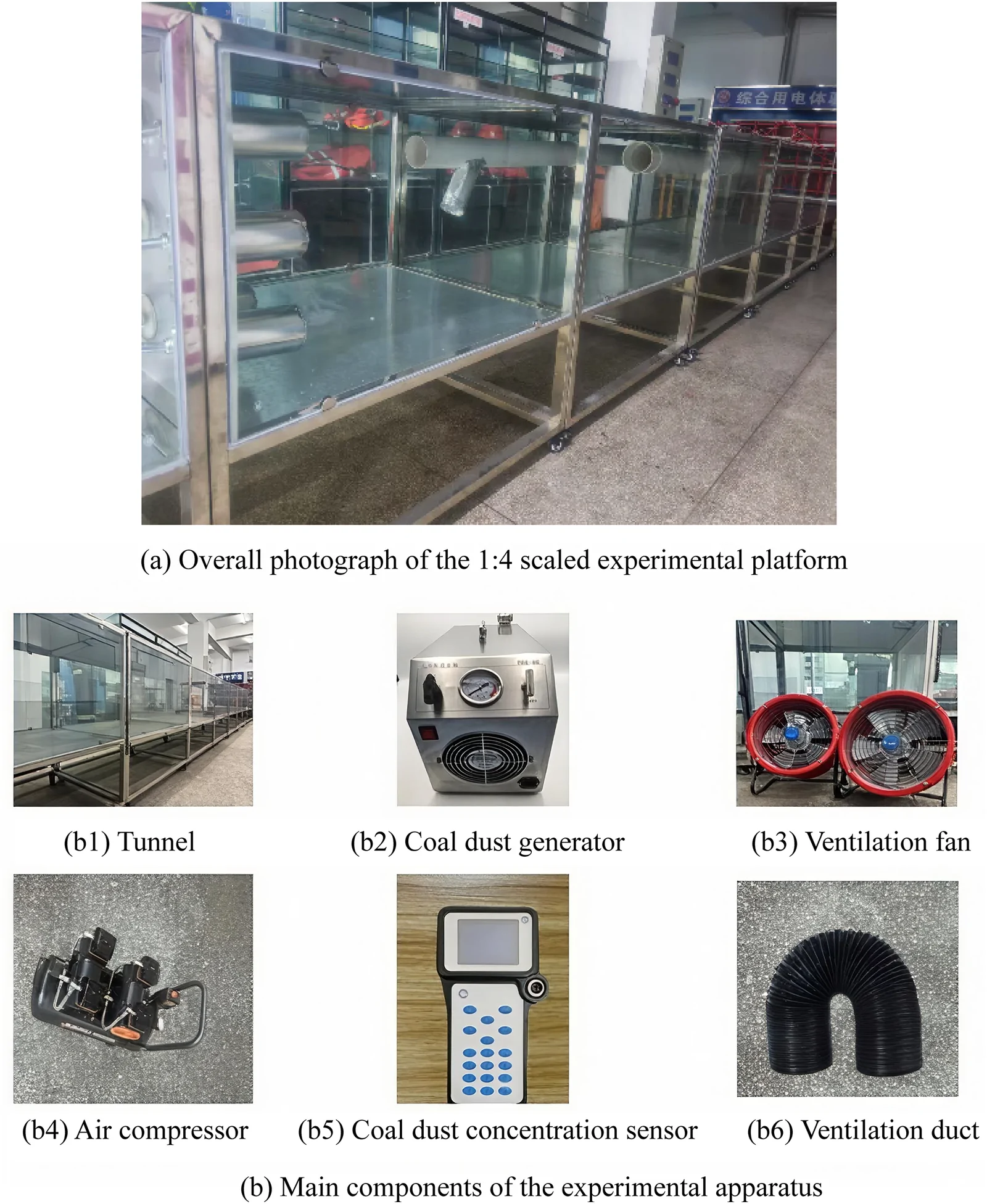
Experimental platform and apparatus layout.

Dust mass concentration sensors were installed at the upper and lower return-air-side corners of the heading face to measure dust mass concentrations under different ventilation conditions. The measurement points and instrument arrangement are shown in Fig 14. The sensors had a measurement range of 0–5000 mg·m^−3^, an accuracy of ±5% FS, and a resolution of 0.1 mg·m^−3^. Each operating condition was tested three times, and the mean of the three measurements was used as the final dust mass concentration. The experimental platform and test procedure were used to validate the numerical simulation results and optimized ventilation scheme and to examine changes in dust mass concentration at the return-air-side corners under different ventilation parameters.

### 4.2. Comparison of experimental results and simulation

To evaluate the reliability of the numerical simulations and response surface optimization results, scaled-model experiments were conducted under three representative ventilation parameter combinations and the optimized condition. For Condition 1, the exhaust duct inlet angle (*A*), exhaust-to-forcing airflow ratio (*B*), exhaust duct inlet diameter (*C*), and forcing duct outlet diameter (*D*) were 45°, 0.4, 0.225 m, and 0.175 m, respectively. For Condition 2, the corresponding values were 60°, 0.3, 0.250 m, and 0.175 m, respectively. For Condition 3, they were 60°, 0.5, 0.250 m, and 0.175 m, respectively. Under the optimized condition, *A*, *B*, *C*, and *D* were set to 75°, 0.3, 0.250 m, and 0.150 m, respectively. Except for these ventilation parameters, the dust-release location, dust-feeding method, total amount of dust released, measurement-point locations, sampling duration, sampling frequency, and other experimental conditions were maintained constant.

**Fig 15:**
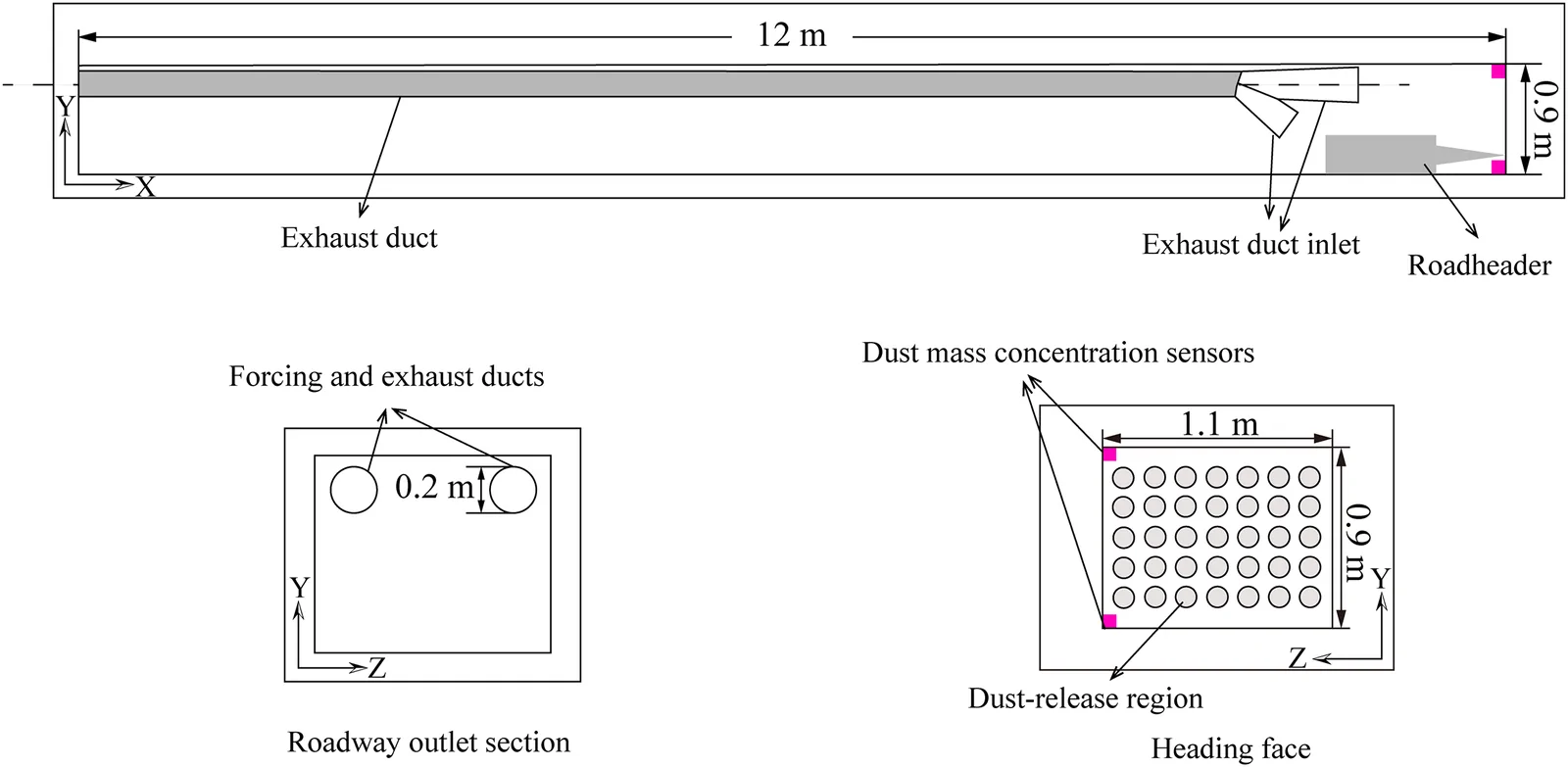
Layout of airflow regulation and dust concentration measurement points.

The experimental apparatus is shown in Fig 14, and the ventilation parameter test arrangement is shown in Fig 15. At the beginning of each test, the forcing and exhaust fans were switched on, and the exhaust duct inlet angle, exhaust-to-forcing airflow ratio, exhaust duct inlet diameter, and forcing duct outlet diameter were adjusted according to the prescribed condition. The ventilation system was then operated for 60 s to allow the airflow field in the roadway to stabilize. Coal dust was subsequently released uniformly across the heading face for 180 s to reproduce dust dispersion from the heading face into the roadway during excavation.

During dust release, dust mass concentration sensors installed at the lower and upper return-air-side corners recorded the dust mass concentrations at *S*_1_ and *S*_2_, respectively. The sampling frequency was 1 Hz, and the sampling duration was 180 s for each test. Each operating condition was tested three times. The time-averaged dust mass concentration was calculated for each test, and the mean of the three repeated tests was used as the final experimental result.

The experimental measurements were quantitatively compared with the corresponding numerical predictions, as summarized in Table 7. The relative error was used to evaluate the agreement between the experimental and numerical results and was calculated as the absolute difference between the simulated dust mass concentration and the mean experimental value divided by the mean experimental value. The measured dust mass concentrations at the lower and upper return-air-side corners showed variation trends consistent with those predicted by the numerical model. Although the simulated dust mass concentrations were generally slightly lower than the experimental values, the relative errors for all operating conditions and measurement points were below 5%. For Conditions 1, 2, 3, and the optimized condition, the relative errors at *S*_1_ were 2.1%, 3.1%, 3.5%, and 4.2%, respectively, whereas those at *S*_2_ were 1.7%, 1.6%, 2.5%, and 2.0%, respectively. The consistent variations in *S*_1_ and *S*_2_ among the different operating conditions indicate that the numerical model can reasonably predict the dust mass concentrations at the lower and upper return-air-side corners.

**Table 7:** Comparison between numerical and experimental results under different ventilation parameter conditions.

Condition	Test point	Numerical resuit (kg/m³)	Experimental results (kg/m³)				Error (%)	SD	CV (%)
			First	Second	Third	Mean			
1	*S*_1_	2.31 × 10^−3^	2.36 × 10^−3^	2.34 × 10^−3^	2.38 × 10^−3^	2.36 × 10^−3^	2.1	0.015	0.63
	*S*_2_	2.88 × 10^−3^	2.93 × 10^−3^	2.91 × 10^−3^	2.95 × 10^−3^	2.93 × 10^−3^	1.7	0.020	0.90
2	*S*_1_	1.85 × 10^−3^	1.89 × 10^−3^	1.93 × 10^−3^	1.91 × 10^−3^	1.91 × 10^−3^	3.1	0.017	0.91
	*S*_2_	2.41 × 10^−3^	2.45 × 10^−3^	2.44 × 10^−3^	2.46 × 10^−3^	2.45 × 10^−3^	1.6	0.010	0.65
3	*S*_1_	2.10 × 10^−3^	2.15 × 10^−3^	2.18 × 10^−3^	2.20 × 10^−3^	2.17 × 10^−3^	3.5	0.018	0.82
	*S*_2_	2.30 × 10^−3^	2.35 × 10^−3^	2.37 × 10^−3^	2.40 × 10^−3^	2.37 × 10^−3^	2.5	0.013	0.55
optimized	*S*_1_	1.94 × 10^−3^	1.99 × 10^−3^	2.02 × 10^−3^	2.07 × 10^−3^	2.03 × 10^−3^	4.2	0.040	1.97
	*S*_2_	2.59 × 10^−3^	2.63 × 10^−3^	2.63 × 10^−3^	2.65 × 10^−3^	2.64 × 10^−3^	2.0	0.012	0.46

To further assess the reliability of the experimental measurements, the potential sources of measurement uncertainty were identified, and experimental repeatability was quantified. The principal sources of uncertainty included sensor accuracy, fluctuations in the dust-release rate, measurement-point positioning errors, and simplifications of the boundary conditions in the scaled model. Experimental repeatability was evaluated using the coefficient of variation, calculated as


$$\begin{array}{c} CV=\frac{\sigma }{\mu }\times 100\% \end{array}$$ (11)

where sigma is the standard deviation of the three repeated measurements and mu is their mean dust mass concentration. As shown in Table 7, the coefficients of variation ranged from 0.63% to 1.97% at *S*_1_ and from 0.46% to 0.90% at *S*_2_. These relatively low values indicate that the repeated measurements had good repeatability.

The small discrepancies between the experimental measurements and numerical predictions may be attributed to geometric simplifications in the scaled model, differences between the laboratory boundary conditions and the actual underground environment, limitations in the measurement-point arrangement, and fluctuations in the dust-generation system. Overall, the agreement between the experimental and numerical results supports the reliability of the numerical method and the effectiveness of the response surface optimization scheme within the investigated parameter ranges.

### 4.3. Discussion

This study combined numerical simulation, response surface methodology (RSM), and scaled-model experiments to investigate dust-control parameters under a long-forcing, short-exhaust hybrid ventilation system at a fully mechanized heading face. In contrast to studies focusing only on individual parameter effects, the present study further considered the coupled effects of the exhaust duct inlet angle, exhaust-to-forcing airflow ratio, exhaust duct inlet diameter, and forcing duct outlet diameter. The dust mass concentrations at the lower and upper return-air-side corners were selected as the optimization objectives to determine a favorable combination of ventilation parameters. The results indicate that dust accumulation at the return-air-side corners under hybrid ventilation conditions is not controlled by a single parameter but is jointly affected by the effective range of the exhaust airflow, the intensity of the forcing-air jet, and the distribution of airflow within the roadway. Therefore, the coupled effects of multiple parameters should be considered when optimizing the ventilation system.

From the perspective of the dust-control mechanism, the reduction in dust mass concentration under the optimized scheme can be attributed mainly to two factors. First, increasing the exhaust duct inlet angle expands the effective range of the exhaust airflow, thereby improving dust removal from the return-air-side corner regions and reducing dust accumulation near the lower return-air-side corner. Second, reducing the forcing duct outlet diameter increases the forcing-air jet velocity, thereby enhancing the dilution and transport of dust particles and improving airflow organization in the corner regions. Under the combined effects of these factors, the optimized ventilation condition reduced the dust mass concentrations at the lower and upper return-air-side corners by approximately 58% and 34%, respectively, compared with the baseline condition.

Previous studies of dust removal under long-forcing, short-exhaust ventilation have also shown that airflow regulation can improve dust control at fully mechanized heading faces. Zheng et al.[31] investigated the effect of the radial-to-axial airflow ratio on airflow uniformity and dust removal in a long-forcing, short-exhaust ventilation system. The dust-removal efficiencies at the operator’s position and at a location 5 m behind the operator increased by 37.7% and 27.7%, respectively. In comparison, the optimized condition in the present study reduced the dust mass concentrations at the lower and upper return-air-side corners by approximately 58% and 34%, respectively. These results indicate that the joint optimization of the exhaust duct inlet angle, exhaust-to-forcing airflow ratio, exhaust duct inlet diameter, and forcing duct outlet diameter can effectively reduce dust accumulation in the return-air-side corner regions. However, direct numerical comparisons of dust-removal efficiency are not appropriate because roadway dimensions, ventilation layouts, dust-release conditions, measurement-point locations, and evaluation criteria differ among studies. The present results primarily demonstrate that the joint adjustment of ventilation parameters targeting local high-concentration zones can substantially reduce dust accumulation at the return-air-side corners and has practical engineering relevance.

The overall dust mass concentration distribution after optimization showed that the high-concentration zones at the return-air-side corners were substantially reduced, with no evident migration of dust toward the central part of the roadway or the main working area. The optimized airflow organization promoted the transport of dust-laden airflow toward the exhaust duct inlets, and no new dust accumulation zones developed in the other principal regions of the roadway. Therefore, the optimized ventilation parameter combination reduced dust mass concentrations at the return-air-side corners without adversely affecting the overall transport and removal of dust within the roadway.

This study has several limitations. In the numerical simulations, airflow in the roadway was treated as incompressible turbulent flow under steady-state conditions, and the actual roadway geometry, boundary conditions, and dust-release process were simplified. The effects of transient excavation activities, fluctuations in the dust-generation rate, variations in temperature and humidity, particle agglomeration, and field disturbances were not fully considered. In addition, the numerical model and scaled-model experimental platform were developed for a specific roadway geometry and long-forcing, short-exhaust ventilation layout. The optimized parameters should therefore be applied cautiously to roadways with different cross-sectional geometries, ventilation duct arrangements, or more complex underground conditions. Future studies could combine field measurements, transient numerical simulations, and multiphysics coupling methods to further evaluate the applicability of the optimized ventilation scheme under different engineering conditions.

## 5. Conclusion

(1) Numerical simulations showed that the lower and upper return-air-side corners of the fully mechanized heading face were the principal dust accumulation zones. Under the baseline ventilation condition, the dust mass concentrations reached 4.52 × 10^−3^ kg·m^−3^ at the lower return-air-side corner and 3.93 × 10^−3^ kg·m^−3^ at the upper return-air-side corner. These results demonstrate the pronounced local accumulation of dust in the return-air-side corner regions.
(2) Response surface methodology (RSM) was employed for multi-parameter optimization by considering the effects of the exhaust duct inlet angle, exhaust-to-forcing airflow ratio, exhaust duct inlet diameter, and forcing duct outlet diameter on the dust mass concentrations. Based on the absolute values of the coded regression coefficients, the effects of the four parameters on the lower return-air-side corner concentration (*S*_1_) followed the order *D* > *A* > *C* > *B*. The corresponding order for the upper return-air-side corner concentration (*S*_2_) was *A* > *B* > *C* > *D*.
(3) The multi-objective optimization produced an optimal parameter combination consisting of an exhaust duct inlet angle of 75°, an exhaust-to-forcing airflow ratio of 0.3, an exhaust duct inlet diameter of 1.0 m, and a forcing duct outlet diameter of 0.6 m. Compared with the baseline condition, this parameter combination reduced the dust mass concentrations at the lower and upper return-air-side corners by approximately 58% and 34%, respectively, substantially reducing the local high-concentration zones.
(4) The main contribution of this study is the establishment of an integrated research framework combining numerical simulation, response surface optimization, and scaled-model experimental validation to address dust accumulation at the return-air-side corners under a long-forcing, short-exhaust hybrid ventilation system. The framework enables the coupled effects of the exhaust duct inlet angle (*A*), exhaust-to-forcing airflow ratio (*B*), exhaust duct inlet diameter (*C*), and forcing duct outlet diameter (*D*) on *S*_1_ and *S*_2_ to be evaluated simultaneously. The 1:4 scaled-model experimental results agreed well with the numerical predictions, with relative errors below 5%. These results support the reliability of the numerical method and optimized ventilation scheme under the investigated operating conditions and provide a reference for dust control and ventilation parameter optimization at similar fully mechanized heading faces.

## Supporting information

S1 File Raw experimental and simulation datasets for the study. (XLSX)

S2 File Metadata and variable definition document for the dataset. (DOCX)
