# Supplementary material for: Research on dust control and ventilation parameter optimization in roadhead mining based on response surface methodology
Source: PLoS One. 2026 Sep 23;21(9):e0338763. doi: 10.1371/journal.pone.0338763 (PMC13600501; doi:10.1371/journal.pone.0338763)
Supplement: Supplementary file 1 [file pone.0338763.s002.docx]

Metadata for Data

This document describes the structure and contents of the file Data.xlsx, which contains the minimal dataset required to replicate the findings reported in the manuscript entitled “Research on Dust Control and Ventilation Parameter Optimization in Roadhead Mining Based on Response Surface Methodology.”

1. Overview

The Data file contains the numerical data underlying the main results reported in the manuscript, including:

the response surface design results used for model development;

the raw repeated experimental measurements used for validation;

the numerical values underlying the graphs presented in the manuscript;

and the summary data for the optimized ventilation condition.

These data are provided in response to the journal’s request for the minimal dataset required to replicate all study findings.

2. File structure

The Excel file Data.xlsx contains four worksheets:

Sheet 1: RSM 29 runs

This sheet contains the 29 runs of the Box-Behnken response surface design used for multi-factor optimization. The variables include the four ventilation parameters and the corresponding dust concentration responses at the two monitoring points.

Sheet 2: Experimental validation raw

This sheet contains the raw repeated experimental measurements used to validate the optimized ventilation condition. For each condition and monitoring point, the numerical simulation value, three replicate experimental values, mean, standard deviation (SD), coefficient of variation (CV), and relative error are provided.

Sheet 3: Plotting data

This sheet contains the numerical values used to generate the graphs presented in the manuscript, including the data underlying the single-factor analysis figures and the grid independence analysis figure.

Sheet 4: Optimal condition summary

This sheet summarizes the predicted, simulated, and experimental results for the optimized ventilation condition.

3. Variable definitions

A: extraction outlet angle (°): extraction outlet angle, in degrees (°)

B: extraction-to-pressure ratio: extraction-to-pressure ratio, dimensionless

C: extraction outlet diameter (m): extraction outlet diameter, in meters (m)

D: pressure duct outlet diameter (m): pressure duct outlet diameter, in meters (m)

S1: lower corner dust (kg/m³): dust concentration at the lower corner of the return air side, in kg/m³

S2: upper corner dust (kg/m³): dust concentration at the upper corner of the return air side, in kg/m³

Simulation value (kg/m³): numerical simulation result, in kg/m³

Replicate 1 (kg/m³) / Replicate 2 (kg/m³) / Replicate 3 (kg/m³): repeated experimental measurements, in kg/m³

Mean (kg/m³): mean of repeated experimental measurements, in kg/m³

SD: standard deviation of repeated measurements

CV (%): coefficient of variation of repeated measurements, expressed as a percentage

Error (%): relative error between simulation and experiment, expressed as a percentage

4. Monitoring locations

Two monitoring points were used in this study:

S1: lower corner of the return air side

S2: upper corner of the return air side

5. Notes on methods

The numerical simulation data were generated based on the CFD model described in the manuscript. Experimental validation was carried out on a 1:4 scaled physical platform, and three repeated experiments were conducted under each operating condition to ensure reliability.

6. Software

Numerical simulations were performed using ANSYS Fluent 2023R1 as described in the manuscript.
